# *In vivo* hypothalamic regional volumetry across the frontotemporal dementia spectrum

**DOI:** 10.1016/j.nicl.2022.103084

**Published:** 2022-06-14

**Authors:** Noah L. Shapiro, Emily G. Todd, Benjamin Billot, David M. Cash, Juan Eugenio Iglesias, Jason D. Warren, Jonathan D. Rohrer, Martina Bocchetta

**Affiliations:** a Dementia Research Centre, Department of Neurodegenerative Disease, UCL Queen Square Institute of Neurology, University College London, UK; b Centre for Medical Image Computing, Department of Medical Physics and Biomedical Engineering, University College London, UK; c UK Dementia Research Institute at UCL, UCL, London, UK; d Martinos Center for Biomedical Imaging, Massachusetts General Hospital and Harvard Medical School, Boston, USA; e Computer Science and Artificial Intelligence Laboratory, Massachusetts Institute of Technology, Boston, USA

**Keywords:** Frontotemporal dementia, Hypothalamus, Volumetry, Magnetic resonance imaging

## Abstract

**Background:**

Frontotemporal dementia (FTD) is a spectrum of diseases characterised by language, behavioural and motor symptoms. Among the different subcortical regions implicated in the FTD symptomatology, the hypothalamus regulates various bodily functions, including eating behaviours which are commonly present across the FTD spectrum. The pattern of specific hypothalamic involvement across the clinical, pathological, and genetic forms of FTD has yet to be fully investigated, and its possible associations with abnormal eating behaviours have yet to be fully explored.

**Methods:**

Using an automated segmentation tool for volumetric T1-weighted MR images, we measured hypothalamic regional volumes in a cohort of 439 patients with FTD (197 behavioural variant FTD [bvFTD]; 7 FTD with associated motor neurone disease [FTD-MND]; 99 semantic variant primary progressive aphasia [svPPA]; 117 non-fluent variant PPA [nfvPPA]; 19 PPA not otherwise specified [PPA-NOS]) and 118 age-matched controls. We compared volumes across the clinical, genetic (29 *MAPT*, 32 *C9orf72*, 23 *GRN*), and pathological diagnoses (61 tauopathy, 40 TDP-43opathy, 4 FUSopathy). We correlated the volumes with presence of abnormal eating behaviours assessed with the revised version of the Cambridge Behavioural Inventory (CBI-R).

**Results:**

On average, FTD patients showed 14% smaller hypothalamic volumes than controls. The groups with the smallest hypothalamic regions were FTD-MND (20%), *MAPT* (25%) and FUS (33%), with differences mainly localised in the anterior and posterior regions. The inferior tuberal region was only significantly smaller in tauopathies (*MAPT* and Pick’s disease) and in TDP-43 type C compared to controls and was the only regions that did not correlate with eating symptoms. PPA-NOS and nfvPPA were the groups with the least frequent eating behaviours and the least hypothalamic involvement.

**Conclusions:**

Abnormal hypothalamic volumes are present in all the FTD forms, but different hypothalamic regions might play a different role in the development of abnormal eating behavioural and metabolic symptoms. These findings might therefore help in the identification of different underlying pathological mechanisms, suggesting the potential use of hypothalamic imaging biomarkers and the research of potential therapeutic targets within the hypothalamic neuropeptides.

## Highlights

• Hypothalamic involvement in seen across the FTD spectrum.
• The hypothalamus was most affected in FTD-MND, *MAPT* and FUS.
• PPA-NOS and nfvPPA showed the least frequent eating behaviours and hypothalamic involvement.
• All hypothalamic regions (except infTub) correlated with aberrant eating behaviours.

## 1 Introduction

Frontotemporal dementia (FTD) is a complex and heterogenous neurodegenerative disease associated with different genetic (Rohrer and Warren, 2011) and pathological causes (Lashley et al., 2015, Mackenzie and Neumann, 2016). Clinically, it typically presents with behavioural, language, or associated motor difficulties (Woollacott and Rohrer, 2016). Given heterogenous symptomatology in FTD, it has been suggested that different neuronal networks might be responsible for the different symptoms observed amongst people with FTD (Warren et al. 2013), although the exact physiological mechanisms by which such symptoms manifest remain to be elucidated.

**Fig. 1:**
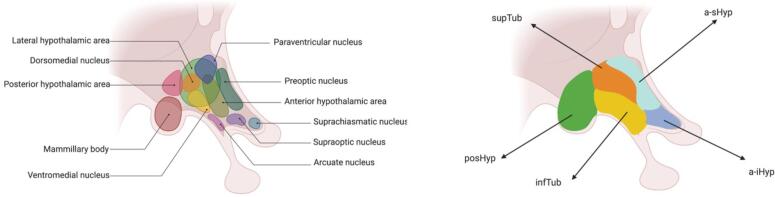
**A schematic representation of the hypothalamus.** Right: the hypothalamus was subdivided into the five subunits: the posterior hypothalamus (posHyp, green), inferior tuberal (infTub, yellow), superior tuberal (supTub, orange), anterior–superior hypothalamus (a-sHyp, light cyan), and anterior-inferior hypothalamus (a-iHyp, light steel blue). Left: the different coloured shapes represent the various hypothalamic nuclei, visualising what nuclei is associated with what subunit. Adapted from “Nuclei of the Hypothalamus”, by BioRender.com (2021). Retrieved from https://app.biorender.com/biorender-templates.

The hypothalamus transmits information from cortical areas to subcortical and brainstem regions, and it is thought to play a role in affecting neuronal networks accounting for autonomic and other dysfunctions seen in people with FTD (Ahmed et al., 2018). The hypothalamus, located on the ventral side of the brain adjacent to the third ventricle and the thalamus (Neudorfer et al., 2020), is comprised of various nuclei (Fig. 1, left panel), each with specialised functions involved in homeostasis of neuroendocrine, behavioural, and autonomic processes (Bocchetta et al., 2021a, Saper and Lowell, 2014, Vercruysse et al., 2018).

A number of studies (Ahmed et al., 2016a, Ahmed et al., 2015, Ahmed et al., 2021a, Dedeene et al., 2019, Perry et al., 2014, Piguet, 2011) have identified deficits in functions regulated by the hypothalamus in some of the FTD forms, with hypothalamic atrophy measured *in vivo* or *post-mortem* (Bocchetta et al., 2015, Bocchetta et al., 2021b, Dedeene et al., 2019, Jones, 2011, Piguet, 2011).

Among its various roles, the hypothalamus regulates aberrant eating behaviours. Such changes are observed in over 60% of patients with behavioural variant FTD (bvFTD) at presentation and in over 75% of people with bvFTD over the course of the disease (Piguet et al., 2009). Changes in eating habits (such as changes in food preference, craving for sweet foods and hyperphagia) are now included as one of the core symptoms for the clinical diagnosis of bvFTD (Rascovsky et al., 2011) and they have been shown to be helpful in discriminating bvFTD from Alzheimer’s disease (Ikeda et al., 2002, Bozeat et al., 2000). Changes in food preference and eating habits are also present in patients with primary progressive aphasia (PPA), particularly the semantic variant (svPPA) (Shinagawa et al., 2009), with rigid eating behaviours and a strong preference for sweet food (Ahmed et al., 2016a, Snowden et al., 2001, Bozeat et al., 2000).

Despite the clear presence of eating disorders in at least some forms of FTD, no study has comprehensively investigated so far the involvement of the hypothalamus and its subregions across the whole spectrum of FTD, and their relationship with eating behaviours. This is due mainly to challenges in measuring hypothalamic regional volumes from magnetic resonance images (MRIs), which so far was possible only by time-consuming manual segmentations (Bocchetta et al., 2015, Schindler et al., 2013). Automatic measurements are now enabled on large cohorts by using our recently developed tool (Billot et al., 2020).

The present study aimed to investigate the detailed patterns of volume changes in the hypothalamic regions in a large retrospective cohort of FTD patients compared to cognitively healthy participants, to determine which hypothalamic regions are involved across the different clinical, genetic, and pathological forms of FTD and how they correlate with aberrant eating behaviours. Results from this study will help to understand the involvement of the hypothalamus across the wide spectrum of FTD, and the relationship between hypothalamic regional abnormalities and presence of eating behaviours. This knowledge will provide further evidence of the involvement of subcortical regions in FTD and of the potential use of imaging markers, together with supporting research into novel therapeutic targets for clinical trials.

## 2 Methods

### 2.1 Participants

The UCL Dementia Research Centre FTD MRI database was reviewed to identify patients diagnosed with a clinical form of FTD (bvFTD (Rascovsky et al., 2011), FTD with associated motor neurone disease [FTD-MND], svPPA, non-fluent variant PPA [nfvPPA] (Gorno-Tempini et al., 2011), PPA not otherwise specified [PPA-NOS] (Harris et al., 2013)) with a volumetric T1-weighted MRI of good quality, as well as cognitively normal controls matched for age and scanner type. We reviewed the MRIs to make sure we excluded individuals with moderate to severe vascular disease or other brain lesions such as tumours. The study was approved by the local ethics committee and written informed consent was obtained from all participants.

The initial selected cohort consisted of 442 FTD patients (bvFTD = 197, svPPA = 100, nfvPPA = 119, PPA-NOS = 19, FTD-MND = 7) and 118 controls (Table 1). Eighty-seven patients carried a pathogenic mutation in one of the genes linked to FTD (Table 2): *MAPT* (n = 29), *C9orf72* (n = 32), *GRN*, (n = 23), and *TBK1* (n = 2) and one patient with FTD-MND had a double mutation in *C9orf72* and *GRN*. For 106 patients, *post-mortem* confirmation of the underlying pathology was available: FUS (n = 4), TDP-43 (n = 40; type A (n = 16), type B (n = 3), type C (n = 21), Tau (n = 62; with Pick's disease (n = 18), with PSP (n = 4), with CBD (n = 9), with GGT1 (n = 2), due to FTD with parkinsonism linked to chromosome 17 FTDP-17 (n = 29)) (Table 2). We excluded subgroups with less than three subjects from the analysis (i.e., *TBK1*, double mutations, tau-GGT1) and findings on groups with less than 5 cases should be taken in careful consideration.

**Table 1:** Demographic and clinical characteristics of the cohort.

	**Controls**	**bvFTD**	**FTD-MND**	**svPPA**	**nfvPPA**	**PPA-NOS**	**p-value**
**N**	118	197	7	100	119	19	
Age, years*	63.2 (8.7)	62.6 (7.8)	66.0 (3.7)	64.2 (7.2)	67.9 (8.6)	63.9 (6.2)	<0.0005
Disease duration, years*	N/A	5.2 (3.3)	4.6 (2.4)	4.7 (2.4)	4.1 (2.2)	3.2 (1.6)	0.004
**Sex, male [%]**	55 [47%]	140 [71%]	4 [57%]	55 [55%]	55 [46%]	12 [63%]	<0.0005
**Scanner type (1.5 T GE /3T Siemens Trio/3T Siemens Prisma)**	46/41/31	83/73/41	4/2/1	53/30/17	47/51/21	4/11/4	0.083

Note: *mean years, standard deviation (SD); Not applicable N/A.

**Table 2:** Distribution of genetic mutation carriers and primary pathologies by the clinical diagnosis.

	**bvFTD**	**FTD-MND**	**svPPA**	**nfvPPA**	**PPA-NOS**
**Mutation carriers**					
***C9orf72***	27	3	–	2	0
***MAPT***	28	0	–	1	0
***GRN***	14	0	–	6	3
***TBK1***	1	–	–	1	–
**Pathologies**					
**Tau**	45	–	4	11	2
Tau-Pick’s	10	–	3	4	1
Tau-CBD	5	–	–	4	–
Tau-PSP	2	–	–	2	–
FTDP-17	28	–	–	1	–
Tau-GGT1	–	–	1	–	1
**TDP-43**	14	1	20	4	1
Type A	11	1	–	3	1
Type B	3	–	–	–	–
Type C	–	–	20	1	–
**FUS**	4	–	–	–	–

A subset of patients (n = 130, Table 3) underwent assessment of behavioural symptoms using the Cambridge Behavioural Inventory Revised version (CBI-R) (Wear et al., 2008). A subset of four questions on the CBI-R addresses the frequency of abnormal eating behaviour scoring 0 for never occurring, 1 occurring a few times per month, 2 occurring a few times per week, 3 occurring daily, and 4 occurring constantly. The questions ask about whether sweet foods are preferred, whether the subject wants to eat the same foods repeatedly, whether their appetite is greater than before, and whether there has been a decline in table manners.

**Table 3:** Behavioural and eating symptoms measured with the CBI-R scale in the patient cohort.

**CBI-R**		**Total (/180)**	**Eating disturbance score (/16)**	**Prefers sweet foods more than before (/4)**	**Wants to eat the same foods repeatedly (/4)**	**Her/his appetite is greater, s/he eats more than before (/4)**	**Table manners are declining e.g., stuffing food into mouth (/4)**
**Clinical diagnosis**							
**bvFTD (n = 59)**	**Mean**	75.80	7.37	2.25	1.92	1.56	1.64
	**SD**	31.28	4.39	1.65	1.47	1.56	1.54
**FTD-MND (n = 3)**	**Mean**	73.67	7.33	2.00	1.67	2.00	1.67
	**SD**	23.03	1.15	2.00	1.53	2.00	1.53
**svPPA (n = 22)**	**Mean**	54.09	4.82	1.64	1.41	0.73	1.05
	**SD**	38.02	5.02	1.73	1.68	1.35	1.4
**nfvPPA (n = 39)**	**Mean**	35.33	2.05	0.69	0.33	0.44	0.59
	**SD**	20.41	2.80	1.15	0.74	0.82	1.29
**PPA-NOS (n = 7)**	**Mean**	32.86	1.86	0.29	0.57	0.86	0.14
	**SD**	29.41	2.12	0.76	1.13	1.21	0.38

**Genetic groups**							

***C9orf72* (n = 16)**	**Mean**	78.94	7.44	1.56	2.19	1.06	2.63
	**SD**	21.57	3.39	1.41	1.33	1.44	1.31
***MAPT* (n = 10)**	**Mean**	59.30	5.60	1.50	1.80	1.20	1.10
	**SD**	30.83	4.79	1.78	1.40	1.48	1.29
***GRN* (n = 10)**	**Mean**	62.00	5.40	1.80	1.00	1.30	1.30
	**SD**	46.19	5.40	1.62	1.49	1.42	1.64

**Pathology groups**							

**TDP-43 (n = 5)**	**Mean**	66.20	4.00	0.80	0.80	0.00	2.40
	**SD**	13.46	2.00	1.30	0.84	0.00	1.82
**Tau (n = 14)**	**Mean**	50.43	4.93	1.36	1.43	1.07	1.07
	**SD**	31.15	4.68	1.78	1.40	1.33	1.44

### 2.2 Imaging

Volumetric T1-weighted MRI scans were obtained using the following scanners and parameters: n = 237 from a 1.5 T Signa (GE Medical systems, Milwaukee, Wisconsin, USA; TR = 12 ms, TI = 650 ms, TE = 5 ms, acquisition matrix = 256×256, thickness = 1.5 mm); n = 208 from a 3 T Trio (Siemens, Erlangen, Germany; TR = 2200 ms, TI = 900 ms, TE = 2.9 ms, acquisition matrix = 256×256, thickness = 1.1 mm); and n = 115 from a 3 T Prisma (Siemens, Erlangen, Germany; TR = 2000 ms, TI = 850 ms, TE = 2.93 ms, acquisition matrix = 256×256, thickness = 1.1 mm) (Table 1 and Supplementary Table 1).

To automatically extract the volumes of the hypothalamus and its five subregions (anterior-inferior [a-iHyp], anterior–superior [a-sHyp], inferior tubular [infTub], superior tubular [supTub] and posterior [posHyp], Fig. 1, right panel and Supplementary Fig. 1), we applied the segmentation tool of Billot et al. (2020) based on a deep convolutional neural network. The whole brain volume was computed by combining the volumes of the white matter and grey matter regions obtained by using the geodesic information flow algorithm (Cardoso et al., 2015), which is based on atlas propagation and label fusion. Total intracranial volume (TIV) was computed using SPM 12 v6470 (Statistical Parametric Mapping, Welcome Trust Centre for Neuroimaging, London, UK) running under MATLAB R2014b (Math Works, MA, USA) (Malone et al. 2015).

Hypothalamic segmentations were examined visually to ensure that the hypothalamus and its subunits were identified correctly by a single trained rater, who referred to a more experienced rater in case of uncertainty and for an additional n = 15 randomly selected sample of segmentations. Three subjects were excluded (one with sporadic svPPA, and 2 with sporadic nfvPPA, of whom one had *post-mortem* confirmation of tau pathology) due to incorrect segmentation, as agreed by two independent evaluators.

### 2.3 Statistical analyses

Statistical analyses were performed using SPSS version 25 (SPSS Inc., Chicago, IL, USA).

Left and right volumes were summed and compared between controls and the patient groups (separately for the clinical, genetic and pathological groups) using a linear regression model (where the groups were considered as between-subject factors), adjusting for age, sex, scanner type, and TIV, with 95% bias-corrected bootstrapped confidence intervals with 1000 repetitions (as the two variables were not normally distributed). P-values were corrected for multiple comparisons (*post-hoc* Bonferroni correction), and α was set at 0.05 after correction.

Spearman's rho correlation analyses were performed separately in each clinical (bvFTD, svPPA, nfvPPA, PPA-NOS, FTD-MND), pathological (tau-opathy and TDP-43-opathy) and genetic subgroup (*C9orf72*, *MAPT*, *GRN* mutation carriers) to test associations between hypothalamic volumes and eating symptoms measured on the CBI-R scale. We considered correction for multiple comparisons using the Benjamini & Hochberg method (Benjamini and Hochberg, 1995) using p = 0.05 for false discovery rate for the correlations. Correlations were not performed in subgroups with less than 3 patients (i.e. FUS and pathology subtypes).

## 3 Results

### 3.1 Demographic data

Overall, there was no significant difference for age (p = 0.270) and scanner type (p = 0.294; including magnetic field strength of the scanner, p = 0.505) between FTD and controls, but there were more men in the patient group than in the control group (p-value = 0.006), especially in the bvFTD group (Table 1).

However, when looking at the specific clinical diagnosis (Table 1), there was a difference in age (p < 0.0005) and disease duration (p = 0.004), driven by nfvPPA being older than svPPA (p = 0.011), bvFTD and controls (p-value < 0.0005), and by bvFTD having a longer disease duration than PPA-NOS (p-value = 0.020).

### 3.2 Behavioural data

#### 3.2.1 Clinical diagnosis

As expected, bvFTD and FTD-MND showed the highest scores for behavioural symptoms in the CBI-R total score (Table 3 and Supplementary Table 2) compared to the language variants, with only bvFTD compared with nfvPPA and PPA-NOS reaching statistically significance (Kruskal-Wallis test, p-value ≤ 0.014).

For the total score for eating disturbance and the individual eating disturbance subscores of the CBI-R, there was a significant difference across all items for the clinical groups (Kruskal-Wallis test, p-value < 0.002). Overall, bvFTD and FTD-MND showed the highest scores for the presence of eating behaviours (scoring 7+ on average), followed by svPPA, and lastly nfvPPA and PPA-NOS, both showing these symptoms the least frequently (Table 3, Supplementary Table 2 and Supplementary Fig. 2). Specifically, bvFTD showed significantly higher values than nfvPPA (mainly driven by decline in table manners, p = 0.001) and PPA-NOS (mainly driven by the increase in appetite, p = 0.003, Supplementary Table 2 and Supplementary Fig. 2). FTD-MND showed higher scores in sweet food preference compared to nfvPPA and PPA-NOS (p-value ≤ 0.034), and in preference in wanting to eat the same foods repeatedly compared to PPA-NOS (p < 0.0005, Supplementary Table 2 and Supplementary Fig. 2).

#### 3.2.2 Genetic diagnosis

*C9orf72* expansion carriers showed high scores in the CBI-R (scoring 79 on average). Overall, *C9orf72* showed the highest scores for the presence of eating behaviours (scoring 7+ on average), followed by *MAPT* and *GRN* mutation carriers (5+) (Table 3, Supplementary Table 2 and Supplementary Fig. 2).

Among mutation carriers, there was a significant difference for table manners (Kruskal-Wallis test, p-value = 0.021), where *C9orf72* expansion carriers tended to show higher scores than both *MAPT* and *GRN* mutation carriers, but the pairwise comparisons did not survive multiple comparison correction (uncorrected p-value ≤ 0.029, Supplementary Table 2).

#### 3.2.3 Pathological diagnosis

Tauopathies scored above 4 on average for the presence of eating behaviours, while TDP-43-opathies scored 4 points (Table 3, Supplementary Table 2 and Supplementary Fig. 2). However, there was no difference between the tau and TDP-43-opathy groups in any scores, probably due to the small sample in these groups. Interestingly, no patients with confirmed TDP-43 at *post-mortem* show any increase in appetite, as all 5 cases scored 0, but there were frequent behaviours related to decline in table manners (Table 3, Supplementary Table 2, and Supplementary Fig. 2).

### 3.3 Brain volumes

As a proxy of disease severity, we compared the whole brain volume across groups and we found that all patients had significantly reduced brain volumes compared to controls (p-value ≤ 0.002, Supplementary Table 3 and Supplementary Table 4), with FTD-MND (8% volumetric difference), *GRN* and FUS (9%) being the subgroups with the highest difference from controls. Among the clinical forms, bvFTD had a smaller brain than svPPA and PPA-NOS, and nfvPPA had a smaller brain than svPPA and PPA-NOS (Supplementary Table 3 and Supplementary Table 4). Among the pathological groups, FUS has a smaller brain than TDP-43, but not from tau. There was no difference across the genetic forms of FTD (Supplementary Table 3 and Supplementary Table 4).

### 3.4 Hypothalamic volumes

#### 3.4.1 Clinical diagnosis

**Fig. 2:**
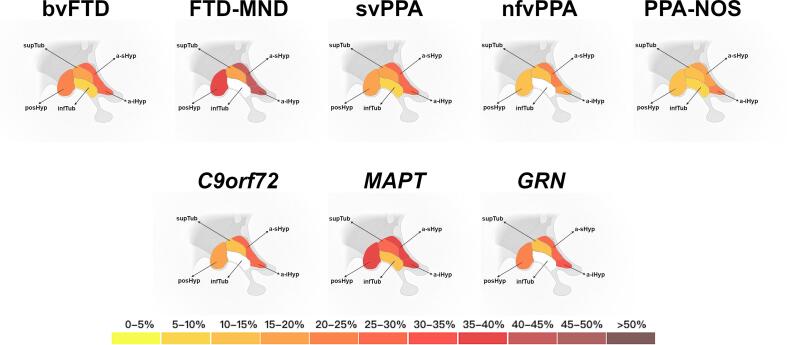
**Pattern of volumetric changes in the hypothalamic regions in the clinical (first row) and genetic (second row) FTD groups. Colour bar denotes the % difference in volume from controls.** Abbreviations: anterior inferior hypothalamus (a-iHyp), anterior superior hypothalamus (a-sHyp), tubular inferior hypothalamus (infTub), tubular superior hypothalamus (supTub), posterior hypothalamus (posHyp). Adapted from “Nuclei of the Hypothalamus”, by BioRender.com (2021). Retrieved from https://app.biorender.com/biorender-templates.

Overall, FTD patients showed significantly smaller hypothalamic volumes (14% difference) compared to controls (p < 0.001, ANCOVA). The clinical group with the highest difference from the controls was FTD-MND (20%), followed by svPPA and bvFTD (15–16%), PPA-NOS (10%) and nfvPPA (8%) (Table 4, Fig. 2 first row, Supplementary Fig. 3A).

The volumes of hypothalamic regions in FTD patients were significantly different from those of controls, except for the infTub region in FTD-MND and nfvPPA (Table 4, Fig. 2).

FTD-MND consistently showed the lowest hypothalamic volumes compared to controls, with volumetric differences of 40–42% in the anterior regions, 35% in the posterior regions, and 17% in the supTub. bvFTD and svPPA patients each showed similar patterns of involvement, the anterior regions being 26–29% smaller than those of controls, the posterior regions showing a 17–22% reduction, the supTub with a 16–17% reduction, and the infTub with a 6–8% reduction (Table 4, Fig. 2).

**Table 4:** **Volumetric comparisons of the hypothalamic regions between the clinical groups and controls.** Volumes are expressed as percentage of TIV. Bold represents a significant difference between each FTD group and controls (reported p-values are adjusted for Bonferroni correction). SD denotes standard deviation. The % difference represents the volumetric difference between each FTD group and controls. Effect size values are partial eta squared.

		**a-iHyp**	**a-sHyp**	**infTub**	**supTub**	**posHyp**	**Whole**
**bvFTD**	Mean	0.00200	0.00256	0.01763	0.01361	0.01261	0.04840
	SD	0.00070	0.00065	0.00265	0.00223	0.00306	0.00762
**FTD-MND**	Mean	0.00165	0.00209	0.01829	0.01355	0.01050	0.04608
	SD	0.00077	0.00092	0.00229	0.00125	0.00198	0.00494
**svPPA**	Mean	0.00202	0.00266	0.01730	0.01365	0.01343	0.04905
	SD	0.00053	0.00060	0.00209	0.00182	0.00272	0.00619
**nfvPPA**	Mean	0.00223	0.00288	0.01864	0.01474	0.01448	0.05296
	SD	0.00057	0.00055	0.00211	0.00159	0.00276	0.00606
**PPA-NOS**	Mean	0.00221	0.00293	0.01783	0.01475	0.01391	0.05163
	SD	0.00065	0.00064	0.00173	0.00170	0.00283	0.00643
**Controls**	Mean	0.00276	0.00360	0.01884	0.01630	0.01615	0.05765
	SD	0.00057	0.00049	0.00151	0.00176	0.00230	0.00491
**% difference**	**bvFTD**	28	29	6	17	22	16
	**FTD-MND**	40	42	3	17	35	20
	**svPPA**	27	26	8	16	17	15
	**nfvPPA**	19	20	1	10	10	8
	**PPA-NOS**	20	19	5	10	14	10
**p-value**	**bvFTD**	**0.001**	**0.001**	**0.001**	**0.001**	**0.001**	**0.001**
	**FTD-MND**	**0.001**	**0.001**	0.553	**0.001**	**0.001**	**0.001**
	**svPPA**	**0.001**	**0.001**	**0.001**	**0.001**	**0.001**	**0.001**
	**nfvPPA**	**0.001**	**0.001**	0.408	**0.001**	**0.001**	**0.001**
	**PPA-NOS**	**0.002**	**0.001**	**0.038**	**0.001**	**0.001**	**0.001**
**effect size**	**bvFTD**	0.171	0.306	0.029	0.207	0.195	0.230
	**FTD-MND**	0.040	0.082	0.001	0.024	0.050	0.041
	**svPPA**	0.120	0.203	0.038	0.148	0.072	0.145
	**nfvPPA**	0.072	0.122	0.001	0.053	0.028	0.047
	**PPA-NOS**	0.021	0.032	0.004	0.016	0.023	0.026

Patients diagnosed with nfvPPA and PPA-NOS showed similar patterns, with a 19–20% reduction in the anterior area, 10–14% reduction in the posterior area, and 10% reduction in the supTub region. infTub region of patients with PPA-NOS had 5% smaller volumes than those of controls.

When comparing the left and right hypothalamic volumes separately, only svPPA and PPA-NOS showed 4-5% more involvement on the left than on the right side (Supplementary Table 5).

When directly comparing the patient groups, bvFTD and FTD-MND showed smaller posterior volumes than all the other language variants (Supplementary Table 6), with FTD-MND showing smaller volumes than bvFTD too. bvFTD showed smaller volumes than nfvPPA in all hypothalamic regions, and smaller volumes in the superior regions compared to PPA-NOS. FTD-MND showed smaller anterior, posterior and supTub volumes than nfvPPA and PPA-NOS, and smaller a-sHyp and posHyp than svPPA (Supplementary Table 6). svPPA showed smaller hypothalamic regions than nfvPPA and smaller superior regions than PPA-NOS.

#### 3.4.2 Genetic diagnosis

The greatest reduction in whole hypothalamic volume was observed in patients with a *MAPT* mutation (25% difference from controls), with involvement mainly localised in the a-sHyp and posterior regions (35–37%), with significantly lower volumes in the supTub (25%) and infTub (13%, p < 0.001) and a symmetric pattern (Supplementary Table 5). Patients with *C9orf72* or *GRN* mutations had a similar pattern of smaller hypothalamic regions than controls, mainly localised in the a-sHyp and posterior regions, but to a less extent than *MAPT* (27–28% and 18–21%, in the a-sHyp and posterior regions respectively), and with smaller volumes in the supTub (10–12%, p < 0.001). With respect to the infTub, both *C9orf72* or *GRN* mutation carriers did not differ significantly from controls (Table 5 and Fig. 2s row, Supplementary Fig. 3B).

**Table 5:** **Volumetric comparisons of the hypothalamic regions between the genetic groups and controls.** Volumes are expressed as percentage of TIV. Bold represents a significant difference between each FTD group and controls (reported p-values are adjusted for Bonferroni correction). SD denotes standard deviation. The % difference represents the volumetric difference between each FTD group and controls. Effect size values are partial eta squared.

		**a-iHyp**	**a-sHyp**	**infTub**	**supTub**	**posHyp**	**Whole**
***C9orf72***	Mean	0.00202	0.00261	0.01879	0.01438	0.01328	0.05109
	SD	0.00069	0.00069	0.00321	0.00249	0.00324	0.00835
***MAPT***	Mean	0.00169	0.00225	0.01641	0.01230	0.01055	0.04320
	SD	0.00047	0.00048	0.00289	0.00247	0.00302	0.00793
***GRN***	Mean	0.00190	0.00263	0.01893	0.01470	0.01272	0.05088
	SD	0.00056	0.00079	0.00199	0.00181	0.00349	0.00663
**% difference**	***C9orf72***	27	28	0	12	18	11
	***MAPT***	39	37	13	25	35	25
	***GRN***	31	27	−1	10	21	12
**p-value**	***C9orf72***	**0.001**	**0.001**	0.979	**0.001**	**0.001**	**0.001**
	***MAPT***	**0.001**	**0.001**	**0.001**	**0.001**	**0.001**	**0.001**
	***GRN***	**0.001**	**0.001**	0.315	**0.003**	**0.001**	**0.001**
**effect size**	***C9orf72***	0.190	0.328	0.000	0.135	0.168	0.182
	***MAPT***	0.285	0.478	0.124	0.392	0.423	0.494
	***GRN***	0.193	0.241	0.007	0.048	0.185	0.125

Overall, patients with a mutation in *C9orf72*, *MAPT*, or *GRN* (n = 84) have a smaller (4%) mean hypothalamic volume than patients with sporadic FTD (n = 352) (p < 0.001, ANCOVA). Patients with a *MAPT* mutation (n = 29) had a smaller (16%) hypothalamic mean volume compared to patients with a *C9orf72* expansion (n = 32) or *GRN* mutation (n = 23) (p < 0.001, ANCOVA). This difference was significant for all regions except for the a-iHyp (Supplementary Table 7).

#### 3.4.3 Pathological diagnosis

**Fig. 3:**
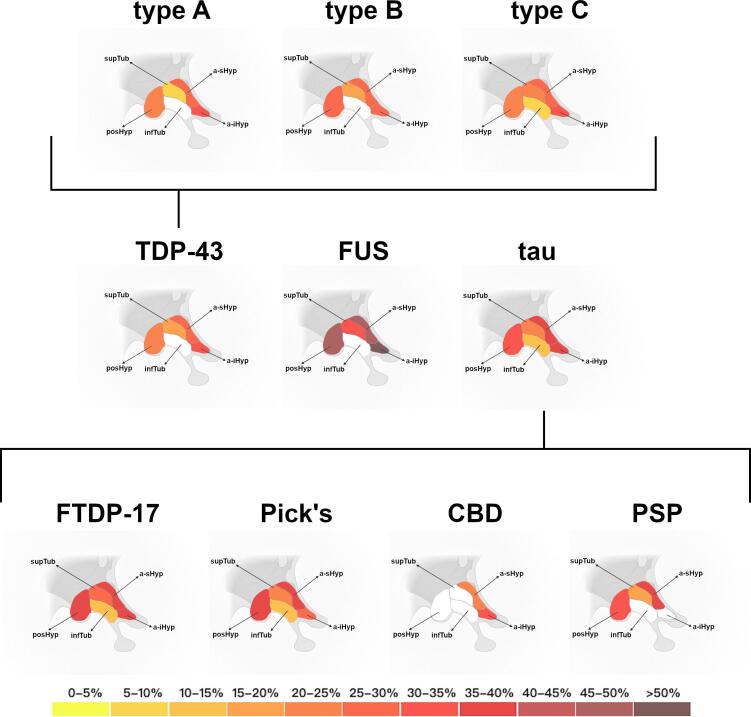
**Pattern of volumetric changes in the hypothalamic regions in the pathological groups. Colour bar denotes the % difference in volume from controls.** Abbreviations: anterior inferior hypothalamus (a-iHyp), anterior superior hypothalamus (a-sHyp), tubular inferior hypothalamus (infTub), tubular superior hypothalamus (supTub), posterior hypothalamus (posHyp). Adapted from “Nuclei of the Hypothalamus”, by BioRender.com (2021). Retrieved from https://app.biorender.com/biorender-templates.

Patients with FUS pathology showed consistently the largest difference from controls for all hypothalamic regions, except for the infTub which was not significant (Table 6 and Fig. 3). Specifically, the largest difference (62%) was in the a-iHyp, followed by the a-sHyp and posterior regions (46–48%) and supTub (31%, p < 0.001). Patients with tauopathies showed significantly smaller volumes in all regions, mainly localised in the anterior (35–37%, p < 0.001) and posterior ones (30%, p < 0.001) (Table 6 and Fig. 3, Supplementary Fig. 3C). Tau was the only pathology group showing significantly smaller infTub volumes compared to controls (10%, p = 0.002).

**Table 6:** **Volumetric comparisons of the hypothalamic regions between the primary pathology groups and controls.** Volumes are expressed as percentage of TIV. Bold represents a significant difference between each FTD group and controls (reported p-values are adjusted for Bonferroni correction). SD denotes standard deviation. The % difference represents the volumetric difference between each FTD group and controls. Effect size values are partial eta squared.

		**a-iHyp**	**a-sHyp**	**infTub**	**supTub**	**posHyp**	**Whole**
**FUS**	Mean	0.00106	0.00186	0.01580	0.01133	0.00877	0.03882
	SD	0.00055	0.00054	0.00257	0.00191	0.00127	0.00589
**TDP-43**	Mean	0.00192	0.00267	0.01798	0.01393	0.01249	0.04899
	SD	0.00048	0.00063	0.00258	0.00179	0.00237	0.00601
**Tau**	Mean	0.00173	0.00232	0.01705	0.01289	0.01127	0.04526
	SD	0.00052	0.00063	0.00256	0.00217	0.00288	0.00736
**% difference**	**FUS**	62	48	16	31	46	33
	**TDP-43**	30	26	5	15	23	15
	**Tau**	37	35	10	21	30	22
**p-value**	**FUS**	**0.001**	**0.001**	0.278	**0.001**	**0.001**	**0.001**
	**TDP-43**	**0.001**	**0.001**	0.217	**0.001**	**0.001**	**0.001**
	**Tau**	**0.001**	**0.001**	**0.002**	**0.001**	**0.001**	**0.001**
**effect size**	**FUS**	0.122	0.144	0.013	0.100	0.145	0.144
	**TDP-43**	0.202	0.276	0.008	0.152	0.172	0.191
	**Tau**	0.339	0.490	0.065	0.351	0.398	0.429

Patients with TDP-43 showed a similar pattern of involvement, with volumetric differences mainly localised in the anterior (26–30%, p < 0.001) and posterior regions (23%, p < 0.001), with only 15% difference from controls in the supTub (Table 6 and Fig. 3).

Patients with abnormal tau pathology had a significantly smaller (6%) hypothalamic mean volume than patients with TDP-43 pathology (p = 0.001, ANCOVA), particularly localised in the a-sHyp, supTub and posHyp (Supplementary Table 8). FUS also showed significantly smaller posterior hypothalamus than both tau and TDP-43 groups, and smaller anterior and supTub volumes than TDP-43 (Supplementary Table 8).

Looking at the specific pathology types among tau, FTDP-17 and tau with Pick’s were the groups with the largest difference and with all subregions significantly smaller than controls (Table 7, Fig. 3). Interestingly, tau with CBD showed involvement only in the anterior regions (20–33%), while tau with PSP shows superior and posterior involvement (17–39%). Among TDP-43 types, type C showed significantly smaller volumes in the tubular regions than type A (Supplementary Table 9). Among tau pathologies, FTDP-17 showed smaller volumes than the other tau forms in the supTub, smaller volumes in the a-sHyp and infTub than tau with CBD and tau with PSP, and smaller posterior volumes than tau with Pick’s and tau with CBD (Supplementary Table 9). Tau with Pick’s showed smaller volumes than tau with CBD in all regions (except a-iHyp), and smaller tuberal regions than tau with PSP. Lastly, tau with PSP showed smaller a-sHyp volumes than tau with CBD (Supplementary Table 9).

**Table 7:** **Volumetric comparisons for the hypothalamic regions between the specific pathology subgroups and controls.** Volumes are expressed as percentage of TIV. Bold represents a significant difference between each FTD group and controls (reported p-values are adjusted for Bonferroni correction). SD denotes standard deviation. The % difference represents the volumetric difference between each FTD group and controls. Effect size values are partial eta squared.

		**a-iHyp**	**a-sHyp**	**infTub**	**supTub**	**posHyp**	**Whole**
**TDP-43 type A**	Mean	0.00193	0.00267	0.01919	0.01504	0.01272	0.05156
	SD	0.00048	0.00063	0.00216	0.00102	0.00217	0.00355
**TDP-43 type B**	Mean	0.00195	0.00254	0.01679	0.01362	0.01157	0.04647
	SD	0.00072	0.00085	0.00386	0.00305	0.00443	0.01288
**TDP-43 type C**	Mean	0.00191	0.00268	0.01724	0.01312	0.01244	0.04739
	SD	0.00047	0.00062	0.00246	0.00168	0.00229	0.00596
**FTDP-17**	Mean	0.00169	0.00225	0.01641	0.01230	0.01055	0.04320
	SD	0.00047	0.00048	0.00289	0.00247	0.00302	0.00793
**Tau-Pick's**	Mean	0.00175	0.00228	0.01703	0.01312	0.01179	0.04597
	SD	0.00044	0.00079	0.00161	0.00168	0.00241	0.00570
**Tau-CBD**	Mean	0.00186	0.00287	0.01878	0.01443	0.01327	0.05122
	SD	0.00058	0.00052	0.00206	0.00161	0.00271	0.00626
**Tau-PSP**	Mean	0.00205	0.00221	0.01805	0.01350	0.01101	0.04682
	SD	0.00087	0.00034	0.00093	0.00134	0.00257	0.00491
**% difference**	**TDP-43 type A**	30	26	−2	8	21	11
	**TDP-43 type B**	29	29	11	16	28	19
	**TDP-43 type C**	31	26	9	20	23	18
	**FTDP-17**	39	37	13	25	35	25
	**Tau-Pick’s**	37	37	10	20	27	20
	**Tau-CBD**	33	20	0	12	18	11
	**Tau-PSP**	26	39	4	17	32	19
**p-value**	**TDP-43 type A**	**0.001**	**0.001**	0.149	**0.002**	**0.001**	**0.001**
	**TDP-43 type B**	**0.001**	**0.001**	0.190	**0.005**	**0.030**	**0.027**
	**TDP-43 type C**	**0.001**	**0.001**	**0.038**	**0.001**	**0.001**	**0.001**
	**FTDP-17**	**0.001**	**0.001**	**0.001**	**0.001**	**0.001**	**0.001**
	**Tau-Pick’s**	**0.001**	**0.001**	**0.006**	**0.001**	**0.001**	**0.001**
	**Tau-CBD**	**0.006**	**0.004**	0.139	0.060	0.079	0.201
	**Tau-PSP**	0.353	**0.001**	0.459	**0.034**	**0.036**	**0.042**
**effect size**	**TDP-43 type A**	0.031	0.032	0.027	0.062	0.058	0.088
	**TDP-43 type B**	0.017	0.006	0.000	0.008	0.018	0.012
	**TDP-43 type C**	0.031	0.036	0.002	0.018	0.069	0.049
	**FTDP-17**	0.013	0.002	0.002	0.000	0.004	0.001
	**Tau-Pick’s**	0.021	0.006	0.001	0.015	0.049	0.033
	**Tau-CBD**	0.031	0.062	0.033	0.054	0.088	0.107
	**Tau-PSP**	0.031	0.015	0.013	0.031	0.029	0.046

### 3.5 Correlations between hypothalamic volumes and eating behaviours

**Fig. 4:**
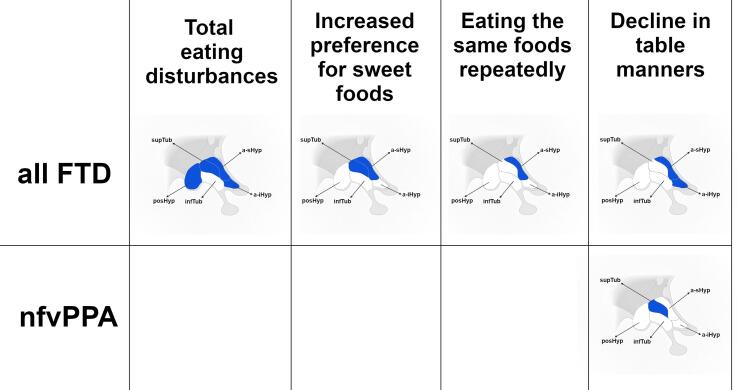
**Pattern of significant negative associations between hypothalamic regions and eating scores on the CBI-R in the FTD patients and those with nfvPPA.** Abbreviations: anterior inferior hypothalamus (a-iHyp), anterior superior hypothalamus (a-sHyp), tubular inferior hypothalamus (infTub), tubular superior hypothalamus (supTub), posterior hypothalamus (posHyp). Adapted from “Nuclei of the Hypothalamus”, by BioRender.com (2021). Retrieved from https://app.biorender.com/biorender-templates.

In all patients with available data, we found that the CBI-R summary scores for the eating behaviours were negatively correlated with all hypothalamic volumes (Spearman’s rho = -0.2/-0.3, p-value ≤ 0.018), except for the infTub (Table 8, Fig. 4). Looking at the individual eating disturbance subscores of the CBI-R, decline in table manners significantly negatively correlated with the anterior subregions (rho = -0.2/-0.3, p-value ≤ 0.016), preference for sweet food negatively correlated with the superior tuberal and anterior areas (rho = -0.2/-0.3, p ≤ 0.008), while wanting to eat the same foods repeatedly negatively correlated with a-sHyp (rho = -0.3, p = 0.001, Table 8, Fig. 4).

**Table 8:** **Spearman’s correlations between eating behaviours measured with the CBI-R scale and hypothalamic volumes.** Bold indicates significantly correlation (p-value < 0.05 after Bonferroni correction for multiple comparisons).

		**a-iHyp**	**a-sHyp**	**infTub**	**supTub**	**posHyp**	**Whole**
**FTD (n = 130)**							
**Total Eating score**	**rho**	**−0.244**	**−0.288**	−0.033	**−0.226**	**−0.207**	**−0.22**
	**p-value**	**0.005**	**0.001**	0.708	**0.010**	**0.018**	**0.012**
**Increased preference for sweet foods**	**rho**	−0.179	**−0.231**	−0.116	**−0.258**	−0.165	**−0.233**
	**p-value**	0.041	**0.008**	0.188	**0.003**	0.061	**0.008**
**Eating the same foods repeatedly**	**rho**	−0.191	**−0.238**	−0.075	−0.188	−0.121	−0.179
	**p-value**	0.030	**0.006**	0.396	0.032	0.169	0.042
**Increased appetite**	**rho**	−0.168	−0.120	0.039	−0.028	−0.149	−0.079
	**p-value**	0.056	0.175	0.663	0.750	0.090	0.373
**Decline in table manners**	**rho**	**−0.211**	**−0.326**	0.081	−0.178	−0.181	−0.139
	**p-value**	**0.016**	**0.000**	0.360	0.043	0.039	0.113

Clinical diagnosis							
**bvFTD (n = 59)**							

**Total Eating score**	**rho**	−0.135	−0.156	0.134	0.044	0.064	0.067
	**p-value**	0.308	0.239	0.313	0.743	0.631	0.612
**Increased preference for sweet foods**	**rho**	−0.117	−0.172	−0.098	−0.167	−0.055	−0.123
	**p-value**	0.376	0.194	0.460	0.207	0.679	0.355
**Eating the same foods repeatedly**	**rho**	−0.053	−0.136	0.066	−0.030	0.160	0.064
	**p-value**	0.689	0.306	0.619	0.821	0.226	0.629
**Increased appetite**	**rho**	−0.152	−0.053	0.164	0.164	0.025	0.097
	**p-value**	0.251	0.691	0.216	0.215	0.851	0.467
**Decline in table manners**	**rho**	−0.061	−0.140	0.262	0.142	0.021	0.140
	**p-value**	0.644	0.291	0.045	0.285	0.875	0.292

**FTD-MND (n = 3)**							

**Total Eating score**	**rho**	−0.866	−0.866	0.866	0.866	0.866	0.866
	**p-value**	0.333	0.333	0.333	0.333	0.333	0.333
**Increased preference for sweet foods**	**rho**	0.500	0.500	−0.500	−0.500	−0.500	−0.500
	**p-value**	0.667	0.667	0.667	0.667	0.667	0.667
**Eating the same foods repeatedly**	**rho**	−0.500	−0.500	0.500	0.500	0.500	0.500
	**p-value**	0.667	0.667	0.667	0.667	0.667	0.667
**Increased appetite**	**rho**	−0.500	−0.500	0.500	0.500	0.500	0.500
	**p-value**	0.667	0.667	0.667	0.667	0.667	0.667
**Decline in table manners**	**rho**	−0.500	−0.500	0.500	0.500	0.500	0.500
	**p-value**	0.667	0.667	0.667	0.667	0.667	0.667

**svPPA (n = 22)**							

**Total Eating score**	**rho**	−0.068	0.149	0.325	0.132	−0.103	0.054
	**p-value**	0.764	0.507	0.140	0.560	0.649	0.810
**Increased preference for sweet foods**	**rho**	−0.050	0.129	0.472	0.080	−0.004	0.151
	**p-value**	0.824	0.568	0.026	0.725	0.987	0.503
**Eating the same foods repeatedly**	**rho**	−0.101	0.105	0.075	0.066	−0.304	−0.117
	**p-value**	0.655	0.641	0.739	0.772	0.169	0.603
**Increased appetite**	**rho**	−0.055	0.105	0.355	0.168	0.095	0.189
	**p-value**	0.806	0.641	0.105	0.454	0.674	0.400
**Decline in table manners**	**rho**	−0.077	−0.073	0.239	0.213	−0.074	0.051
	**p-value**	0.734	0.748	0.284	0.340	0.744	0.823

**nfvPPA (n = 39)**							

**Total Eating score**	**rho**	−0.154	−0.133	−0.090	−0.332	−0.249	−0.276
	**p-value**	0.348	0.420	0.587	0.039	0.127	0.089
**Increased preference for sweet foods**	**rho**	−0.112	−0.046	−0.139	−0.151	−0.085	−0.144
	**p-value**	0.498	0.783	0.400	0.360	0.605	0.382
**Eating the same foods repeatedly**	**rho**	−0.041	0.005	−0.169	−0.201	−0.158	−0.203
	**p-value**	0.805	0.977	0.304	0.220	0.336	0.215
**Increased appetite**	**rho**	0.028	0.079	−0.099	−0.196	−0.066	−0.101
	**p-value**	0.865	0.631	0.550	0.232	0.689	0.540
**Decline in table manners**	**rho**	−0.179	−0.316	−0.024	**−0.494**	−0.171	−0.234
	**p-value**	0.276	0.050	0.886	**0.001**	0.298	0.152

**PPA-NOS (n = 7)**							

**Total Eating score**	**rho**	0.145	−0.509	−0.109	0.582	−0.036	−0.164
	**p-value**	0.756	0.243	0.816	0.170	0.938	0.726
**Increased preference for sweet foods**	**rho**	0.204	−0.408	0.204	0.612	0.204	0.000
	**p-value**	0.661	0.363	0.661	0.144	0.661	1.000
**Eating the same foods repeatedly**	**rho**	0.045	−0.089	0.445	0.535	0.445	0.267
	**p-value**	0.924	0.849	0.317	0.216	0.317	0.562
**Increased appetite**	**rho**	0.355	−0.236	−0.315	0.512	−0.079	−0.099
	**p-value**	0.435	0.610	0.491	0.240	0.867	0.834
**Decline in table manners**	**rho**	−0.612	−0.612	−0.204	−0.612	−0.612	−0.612
	**p-value**	0.144	0.144	0.661	0.144	0.144	0.144

Genetic groups							
***C9orf72* (n = 16)**							

**Total Eating score**	**rho**	−0.021	0.157	0.128	0.313	0.077	0.156
	**p-value**	0.939	0.561	0.638	0.238	0.777	0.565
**Increased preference for sweet foods**	**rho**	−0.021	−0.032	−0.186	−0.169	0.109	−0.076
	**p-value**	0.938	0.907	0.491	0.531	0.688	0.781
**Eating the same foods repeatedly**	**rho**	0.371	0.332	0.293	0.485	0.451	0.553
	**p-value**	0.157	0.209	0.271	0.057	0.080	0.026
**Increased appetite**	**rho**	−0.063	0.199	−0.047	0.444	−0.034	0.088
	**p-value**	0.815	0.461	0.862	0.085	0.900	0.746
**Decline in table manners**	**rho**	−0.409	−0.298	0.394	−0.015	−0.212	−0.081
	**p-value**	0.115	0.262	0.131	0.955	0.432	0.767

***MAPT* (n = 10)**							

**Total Eating score**	**rho**	−0.438	−0.438	0.292	−0.140	−0.164	−0.109
	**p-value**	0.206	0.206	0.413	0.700	0.650	0.763
**Increased preference for sweet foods**	**rho**	−0.273	−0.221	0.391	−0.020	0.130	0.104
	**p-value**	0.445	0.539	0.264	0.957	0.720	0.775
**Eating the same foods repeatedly**	**rho**	−0.393	−0.362	0.368	0.168	0.175	0.212
	**p-value**	0.262	0.305	0.296	0.642	0.630	0.557
**Increased appetite**	**rho**	−0.311	−0.285	0.253	−0.195	−0.409	−0.162
	**p-value**	0.381	0.424	0.481	0.590	0.241	0.654
**Decline in table manners**	**rho**	−0.420	−0.502	−0.299	−0.68	−0.407	−0.763
	**p-value**	0.227	0.139	0.402	0.030	0.243	0.010

***GRN* (n = 10)**							

**Total Eating score**	**rho**	−0.167	−0.446	−0.334	−0.452	−0.291	−0.557
	**p-value**	0.644	0.196	0.345	0.190	0.415	0.094
**Increased preference for sweet foods**	**rho**	−0.129	−0.291	−0.226	−0.388	−0.226	−0.453
	**p-value**	0.722	0.415	0.530	0.268	0.530	0.189
**Eating the same foods repeatedly**	**rho**	−0.239	−0.512	−0.055	−0.362	−0.280	−0.478
	**p-value**	0.506	0.130	0.881	0.304	0.433	0.162
**Increased appetite**	**rho**	−0.082	−0.371	−0.365	−0.346	−0.227	−0.459
	**p-value**	0.822	0.291	0.300	0.327	0.529	0.182
**Decline in table manners**	**rho**	−0.234	−0.547	−0.202	−0.482	−0.365	−0.592
	**p-value**	0.515	0.102	0.576	0.159	0.300	0.071

Pathology groups							
**TDP-43 (n = 5)**							

**Total Eating score**	**rho**	0.359	−0.154	0.975	0.359	0.051	0.821
	**p-value**	0.553	0.805	0.005	0.553	0.935	0.089
**Increased preference for sweet foods**	**rho**	−0.671	0.224	−0.112	0.447	−0.224	0.112
	**p-value**	0.215	0.718	0.858	0.450	0.718	0.858
**Eating the same foods repeatedly**	**rho**	0.527	−0.264	0.949	0.264	0.211	0.791
	**p-value**	0.361	0.668	0.014	0.668	0.734	0.111
**Increased appetite**	**rho**	–	–	–	–	–	–
	**p-value**	–	–	–	–	–	–
**Decline in table manners**	**rho**	0.462	−0.410	0.718	0.205	0.154	0.564
	**p-value**	0.434	0.493	0.172	0.741	0.805	0.322

**Tau (n = 14)**							

**Total Eating score**	**rho**	−0.179	−0.237	0.004	−0.137	0.095	−0.031
	**p-value**	0.539	0.415	0.988	0.640	0.746	0.916
**Increased preference for sweet foods**	**rho**	−0.078	−0.123	0.125	−0.059	0.218	0.088
	**p-value**	0.790	0.676	0.670	0.842	0.454	0.764
**Eating the same foods repeatedly**	**rho**	−0.284	−0.388	0.061	−0.070	0.093	−0.043
	**p-value**	0.325	0.170	0.835	0.811	0.752	0.884
**Increased appetite**	**rho**	−0.168	−0.139	0.227	−0.035	−0.295	−0.057
	**p-value**	0.567	0.635	0.436	0.904	0.305	0.847
**Decline in table manners**	**rho**	−0.173	−0.286	−0.390	−0.520	−0.031	−0.437
	**p-value**	0.555	0.321	0.168	0.056	0.917	0.118

Among the clinical subgroups, in nfvPPA patients, there was a significant correlation between decline in table manners and lower volumes in the supTub (rho = -0.5, p-values = 0.001, Table 8, Fig. 4).

No other correlations survived multiple comparison correction across genetic and pathology groups, probably related to the relatively small groups for which the data were available.

## 4 Discussion

By using a newly developed automated segmentation tool for MRI, the present study investigated *in vivo* the involvement of hypothalamic regions in a large well-characterised cohort of FTD patients.

We found that all groups were showing abnormal volumes of the hypothalamus, with relatively symmetric pattern of volume differences, except for svPPA and PPA-NOS, where the left side was more affected than the right, as expected from these asymmetric diseases. All the hypothalamic regions (except for the infTub) correlated with aberrant eating behaviours in the group of patients, suggesting that the hypothalamus might be related to eating and perhaps metabolic symptoms across the FTD spectrum (Ahmed et al., 2016b).

The hypothalamic involvement was mainly localised in the anterior and posterior regions, which include nuclei regulating the appetite via neuropeptide receptors (i.e. paraventricular nuclei, lateral hypothalamic area) (Neudorfer et al., 2020, Parker and Bloom, 2012, Jones, 2011). In particular, the lateral hypothalamus (included in the superior and posterior subregions analysed here) plays a role in the regulation of feeding, arousal, energy balance, reward and motivated behaviours via complex interactions with different brain networks (Arrigoni et al., 2019, Vercruysse et al., 2018), typically affected across the FTD spectrum. The common involvement of the anterior and posterior regions across the FTD spectrum could be related to different aspects of metabolic homeostasis regulation, rather than the result of a common underlying mechanism. For instance, abnormal feeding and reward processing could be linked with atrophy in the posterior hypothalamus via the limbic regions, whilst dysregulation of circadian rhythms, thermoregulation, and sleep-wake cycle with atrophy in the anterior regions (Arrigoni et al., 2019, Vercruysse et al., 2018, Saper and Lowell, 2014). Different neuronal correlates may suggest a complex interaction between eating behaviour, autonomic functions, and energy homeostasis across FTD (Ahmed et al., 2017, Ahmed et al., 2021a, Warren and Clark, 2017). This might also explain the different associations that we found between specific eating behaviours, such as the correlation between the anterior regions and decline in table manners, and the association between superior regions and rigidity in food preference, especially for sweet food.

The lack of significant correlations between eating behaviours and the infTub, together with the fact that this region was relatively spared (except for tauopathies as discussed later on), seems to suggest that the nuclei in this region are not linked with the emergence of the eating behaviours seen across FTD.

*Clinical diagnosis.* Among the clinical groups, the smallest hypothalamic regions were found in FTD-MND, followed by bvFTD and svPPA. In line with both the neuroanatomical and behavioural profiles emerging in this study, these forms have been typically reported to present with abnormal eating symptoms (Piguet et al., 2009, Ikeda et al., 2002, Bozeat et al., 2000, Shinagawa et al., 2009, Ahmed et al., 2016a, Snowden et al., 2001, Ahmed et al., 2021b), while nfvPPA and PPA-NOS rarely present with them. Both FTD-MND and bvFTD showed high scores in all CBI-R eating items, whilst svPPA showed rather rigid eating behaviours and preference for sweets, rather than increase in appetite, as previously shown (Shinagawa et al., 2009, Ahmed et al., 2016a, Snowden et al., 2001, Bozeat et al., 2000).

FTD-MND showed the most severe volumetric reduction, which might be explained by the severity of the disease (but not necessary duration), as also supported by the fact that this group showed the smallest overall brain volume. However, whilst the overall brain difference from controls was only 8%, the hypothalamus showed 20% difference on average (with regions surpassing 40%). This might suggest that the hypothalamus is one of the first regions to be affected by neurodegeneration in FTD-MND (Ahmed et al., 2021b, Lillo et al., 2012), but this needs further investigations on larger samples, and we should consider our results as an exploratory analysis.

Previous studies in bvFTD (Piguet, 2011, Bocchetta et al., 2015) have reported smaller hypothalamus (especially in the superior and posterior regions) linked with severe eating disturbances: hyperorality and dietary changes are likely the result of dysfunctional neuronal networks in conjunction with appetite-stimulating pathways controlled by the hypothalamus and other structures (Ahmed et al., 2016a, Arrigoni et al., 2019). Moreover, both bvFTD and svPPA patients showed altered eating behaviours and autonomic dysfunction (Ahmed et al., 2014, Ahmed et al., 2015). Whilst in bvFTD the increased caloric intake could be related to changes in the reward network (hypothalamus, thalamus and other limbic regions) (Ahmed et al., 2021a, Perry et al., 2014), symptoms in svPPA could be due to impairments in the semantic knowledge of different foods, via connections between the hypothalamus and the anterior and mediotemporal lobe (Ahmed et al., 2021a, Omar et al., 2013, Vignando et al., 2019).

The involvement of the hypothalamus in bvFTD, FTD-MND and svPPA could be also related to its role with and connections to the abnormal orbitofrontal-insular-striatal brain network (Woolley et al., 2007, Whitwell et al., 2007, Bozeat et al., 2000), and specifically with the insula, often severely affected in all these three forms (Ahmed et al., 2021b, Whitwell, 2019). Additionally, the suprachiasmatic nucleus, included in the a-iHyp subunit, largely regulates the sleep cycles, which have been found disrupted in bvFTD patients (Bonakis et al., 2014, Sani et al., 2019).

*Genetic diagnosis.* Among the genetic groups, and despite the relatively small volumetric difference of the whole brain from controls, *MAPT* mutation carriers showed the largest difference in the hypothalamic volumes from controls, and involvement of all regions of the hypothalamus, in line with data previously shown in a large genetic cohort (Bocchetta et al., 2021a). Once again, the pattern of hypothalamic changes was mainly localised in the anterior and posterior regions of the hypothalamus. Interestingly, the posterior region includes the mammillary bodies and the tuberomammillary nuclei, which are connected with limbic regions such as the amygdala, hippocampus and accumbens nucleus, typically involved in *MAPT* (Bocchetta et al., 2021a) to regulate sleep and wakefulness, food-reward and arousal stability (Fujita et al., 2017). The involvement of the hypothalamus in *C9orf72* and *GRN* mutation carriers is in line with what previously reported (Bocchetta et al., 2021a), which has found that only in *C9orf72* expansion carriers the changes are measurable before the onset of clinical symptoms. A *post-mortem* study in *C9orf72* expansion carriers with symptoms in the ALS-FTD spectrum has found dipeptide repeat protein inclusions in the suprachiasmatic nucleus (part of the a-iHyp subunit and related to circadian sleep-wake regulation), but no TDP-43 deposition (Dedeene et al., 2019). Although not showing disproportionately smaller hypothalamic compared to the other groups, *C9orf72* expansion carriers typically showed altered processing of pain and temperature awareness (Fletcher et al., 2015): these complex symptoms are likely dependent on the hypothalamic connections with other structures, including the thalamus (Bocchetta et al., 2020).

*Pathological diagnosis.* The largest difference from controls was found in FUS. Limited research is available into the hypothalamus in FUS, however the involvement of the posterior regions might be related to their connections with structures in the temporal lobe, where FUS-related pathology was found *post-mortem* (Lashley et al., 2011). FUS was also the group with the most severe volume brain loss, and we cannot exclude that the only 4 patients in the group were at a late stage of the disease (despite being only 3.1 years on average since their diagnosis), and therefore the neurodegeneration might have been widespread and not specifically localised in the hypothalamus. Unfortunately, we did not have data on the eating behaviours available for these patients to be correlated with the volumes. Further studies are needed to clarify the relationship between FUS and the hypothalamus in FTD.

Tauopathies (both genetic and pathological groups) showed significantly smaller volumes than TDP-43opathies, involving the whole hypothalamus. In particular, the infTub region was affected only in the tauopathies, but not in the TDP-43 nor FUS groups. The infTub subunit includes the arcuate, ventromedial, supraoptic, lateral tubular and tuberomamillary nuclei (Bocchetta et al., 2015, Billot et al., 2020). Previous *post-mortem* studies have identified abnormal TDP-43 inclusions in the anterior, superior and posterior hypothalamus, but not in the arcuate and supraoptic nuclei, which are both included in the infTub (Cykowski et al., 2016, Cykowski et al., 2014). The arcuate is a target of metabolic and hormonal signals from the periphery and linked with the paraventricular nucleus (Parker and Bloom, 2012, Joly-Amado et al., 2014, Ahmed et al., 2021a). The lack of involvement in this nucleus might suggest differential pathological mechanisms between tauopathies and TDP-43-opathies in the regulation of eating behaviours: this information is particularly relevant when thinking of possible therapeutic targets for symptom management, which might work for one subtype but not for another. The only other group with involvement in the infTub was TDP-43 type C (and the corresponded svPPA clinical group), but in this study we did not find significant association between infTub and eating symptoms. Perhaps the reduced volumes of infTub in svPPA/TDP-43 type C are linked with other metabolic symptoms (such as energy expenditure) rather than the eating behaviours measured with the CBI-R scale.

The largest volumetric difference (mainly in the posterior hypothalamus) in tau compared to TDP-43 is in line with previous findings (Vercruysse et al., 2018), but in contrast with others (Piguet, 2011). Interestingly, FTD-MND (the clinical form with the smallest hypothalamus) is typically characterised by TDP-43 pathology rather than tau (Tan et al., 2015), whilst *MAPT* (causing tau pathology) was more affected than *C9orf72* and *GRN*, both presenting with TDP-43 accumulation at *post-mortem*. Perhaps there is an interacting effect of genetic, type of pathology and environmental factors in these apparently conflicting results, which should be further explored.

There was a differential involvement across the tau specific pathologies, with tau due to *MAPT* mutations and tau with Pick’s being the subtypes with the largest differences from controls. A study has found that the lateral tuberal nucleus (part of the infTub) was severely affected in Pick’s disease (Munoz et al., 2003). Tau with CBD and tau with PSP were the groups with the least hypothalamic involvement, with differences in the anterior regions in tau-CBD, and in the superior and posterior areas in tau-PSP. These data suggest that the regulation of feeding and autonomic system via the hypothalamus might be different across specific pathologies in FTD, and further studies are needed to better understand their relationships.

*Limitations.* There are a number of limitations in this study, including the small sample size for some of the subgroups and particularly FUS and FTD-MND, which should be considered with extreme caution and only as exploratory analyses. Moreover, we did not have data available on the whole cohort for behavioural nor any neuropeptide measures, to be able to further explore the associations between the hypothalamic volumes and the specific FTD symptomatology. Moreover, the assessment of eating behaviours in the CBI-R is limited to 4 items, while the reality of such behaviours is more complex and varied, and it requires further exploration. An example of a more comprehensive measure of eating behaviour abnormalities is the Appetite and Eating Habits Questionnaire (Ahmed et al., 2014, Ikeda et al., 2002).

Different image resolutions could have had an effect on the segmentation accuracy, especially for the anterior regions, which were also the ones with the lowest reliability, as described in the original paper (Billot et al., 2020). To reduce this potential confounding effect, we performed a careful visual check for quality on all subjects and excluded those with poor performance; we also verified that the patient and control groups were equally distributed among scanner types, which were also included as a covariate in the analysis, adjusting for the effect of different acquisition protocols. However, this is a large cohort and the first study to investigate the regional hypothalamic impairment and to show the intrinsic link between FTD and hypothalamic damage localized to regions regulating food intake, reward and circadian rhythms.

## 5 Conclusion and future directions

This study shows that reduced hypothalamic volumes are present across the FTD spectrum and can be measured *in vivo*. Furthermore, they are linked with abnormal eating behaviours. These findings provide further evidence of the role of subcortical structures in the symptomatology of FTD; moreover, the differential involvement of the hypothalamus across FTD forms has important clinical implications, not only for the use of specific imaging biomarkers in trial design, but also in the quest for therapeutic targets to manage behavioural and metabolic symptoms, such as specific neuropeptides. Genetic mutations and different types of pathology seem to have a differential impact on the hypothalamus, although future studies are needed to further elucidate the differential effects of proteinopathies on the hypothalamic volume, together with longitudinal measures to understand at what disease stage the hypothalamus become involved, how fast these changes are occurring, and to investigate the role of different neuropeptides. Symptoms related to the hypothalamus are intrinsically complex, and despite having highlighted in this study the involvement of such structure across the FTD spectrum and potential link with eating behavioural symptoms, future studies are needed to better understand the interactions of the hypothalamus with the other brain networks. In this contest, it will be important to investigate the spreading of neurodegeneration and abnormal protein accumulation in these networks across the clinical forms due to different pathologies, and the evolution of potential lateralization and hypothalamic involvement.

## CRediT authorship contribution statement

**Noah L. Shapiro:** Writing – original draft, Formal analysis, Visualization. **Emily G. Todd:** Writing – review & editing, Data curation. **Benjamin Billot:** Writing – review & editing, Software, Methodology. **David M. Cash:** Writing – review & editing, Data curation. **Juan Eugenio Iglesias:** Writing – review & editing, Software, Methodology. **Jason D. Warren:** Writing – review & editing, Conceptualization, Data curation. **Jonathan D. Rohrer:** Writing – review & editing, Conceptualization, Data curation. **Martina Bocchetta:** Writing – review & editing, Supervision, Conceptualization, Formal analysis, Visualization, Methodology.

## Declaration of Competing Interest

The authors declare that they have no known competing financial interests or personal relationships that could have appeared to influence the work reported in this paper.
