# Supplementary material for: In vivo hypothalamic regional volumetry across the frontotemporal dementia spectrum
Source: Neuroimage Clin. 2022 Jun 14;35:103084. doi: 10.1016/j.nicl.2022.103084 (PMC9218583; doi:10.1016/j.nicl.2022.103084)
Supplement: Supplementary data 1 [file mmc1.docx]

**Supplementary Table 1: Distribution of the magnetic field strength of the scanners across groups.**

|  | | **1.5T** | **3T** |
| --- | --- | --- | --- |
| **Controls** | | 46 | 72 |
| **Clinical group** | **bvFTD** | 83 | 114 |
|  | **FTD-MND** | 4 | 3 |
|  | **svPPA** | 53 | 46 |
|  | **nfvPPA** | 45 | 72 |
|  | **PPA-NOS** | 4 | 15 |
| **Genetic groups** | ***C9orf72*** | 10 | 23 |
|  | ***MAPT*** | 13 | 16 |
|  | ***GRN*** | 8 | 15 |
| **Pathological groups** | **FUS** | 2 | 2 |
|  | **TDP-43** | 27 | 13 |
|  | **TAU** | 36 | 25 |

**Supplementary Table 2: Pairwise comparisons (Kruskal-Wallis test) for the CBI-R scores between each FTD groups.** Values denoted p-values after Bonferroni correction.

|  | **CBI-R** | **Total (/180)** | **Eating disturbance score (/16)** | **Prefers sweet foods more than before (/4)** | **Wants to eat the same foods repeatedly (/4)** | **Her/his appetite is greater, s/he eats more than before (/4)** | **Table manners are declining e.g., stuffing food into mouth (/4)** |
| --- | --- | --- | --- | --- | --- | --- | --- |
| **Clinical diagnosis** | | | | | | | |
| **bvFTD** | **FTD-MND** | 1.000 | 1.000 | 1.000 | 1.000 | 1.000 | 1.000 |
|  | **svPPA** | 0.064 | 0.257 | 1.000 | 1.000 | 1.000 | 1.000 |
|  | **nfvPPA** | **0.000** | **0.038** | 1.000 | 1.000 | 0.164 | **0.001** |
|  | **PPA-NOS** | **0.014** | **0.000** | 1.000 | 1.000 | **0.003** | 0.092 |
| **FTD-MND** | **svPPA** | 1.000 | 1.000 | 1.000 | 1.000 | 1.000 | 1.000 |
|  | **nfvPPA** | 0.530 | 0.747 | **0.000** | 0.272 | 1.000 | 1.000 |
|  | **PPA-NOS** | 0.748 | 0.334 | **0.034** | **0.000** | 1.000 | 1.000 |
| **svPPA** | **nfvPPA** | 0.817 | 1.000 | 0.560 | 1.000 | 1.000 | 1.000 |
|  | **PPA-NOS** | 1.000 | 0.283 | 0.791 | 0.111 | 1.000 | 1.000 |
| **nfvPPA** | **PPA-NOS** | 1.000 | 1.000 | 1.000 | 1.000 | 1.000 | 1.000 |
| **Genetic diagnosis** | | | | | | | |
| ***C9orf72*** | ***MAPT*** | 1.000 | 1.000 | 1.000 | 1.000 | 1.000 | 0.092 |
|  | ***GRN*** | 1.000 | 1.000 | 1.000 | 1.000 | 1.000 | 0.173 |
| ***MAPT*** | ***GRN*** | 1.000 | 1.000 | 1.000 | 1.000 | 1.000 | 1.000 |
| **Pathological diagnosis** | | | | | | | |
| **Tau** | **TDP-43** | 0.228 | 0.926 | 0.641 | 0.442 | 0.060 | 0.145 |

**Supplementary Table 3: Mean and standard deviation (SD) of the whole brain volumes for the clinical, genetic and pathological groups and controls.** Volumes are expressed as percentage of TIV.

|  |  | **Mean** | **SD** |
| --- | --- | --- | --- |
|  | **Control** | 79.0 | 3.1 |
| **Clinical** | **bvFTD** | 73.5 | 4.2 |
|  | **FTD-MND** | 72.8 | 3.4 |
|  | **svPPA** | 75.6 | 2.9 |
|  | **nfvPPA** | 73.8 | 3.6 |
|  | **PPA-NOS** | 75.5 | 2.7 |
| **Genetic** | ***C9orf72*** | 73.2 | 4.1 |
|  | ***MAPT*** | 75.2 | 3.3 |
|  | ***GRN*** | 71.5 | 5.6 |
| **Pathology** | **FUS** | 72.3 | 2.7 |
|  | **TDP-43** | 74.8 | 3.4 |
|  | **TAU** | 73.9 | 3.3 |

**Supplementary Table 4: Volumetric comparisons of the whole brain between the FTD groups and controls.** Bold represents a significant difference between each pair of groups after Bonferroni correction. The % difference represents the volumetric difference between each FTD group and controls.

|  | | **% difference** | **Mean difference (mm^3^)** | **p-value** | **95% Confidence Interval** | |
| --- | --- | --- | --- | --- | --- | --- |
|  |  |  |  |  | **Lower** | **Upper** |
| **Clinical diagnosis** | | | | | | |
| **Control** | **bvFTD** | **7.0** | **76979.1** | **<.001** | **65911.3** | **88060.6** |
|  | **FTD-MND** | **7.9** | **83082.0** | **0.002** | **34677.6** | **125323.5** |
|  | **svPPA** | **4.4** | **46628.0** | **<.001** | **35831.1** | **58080.0** |
|  | **nfvPPA** | **6.6** | **65755.5** | **<.001** | **53708.9** | **77292.1** |
|  | **PPA-NOS** | **4.5** | **40642.6** | **<.001** | **24167.5** | **57636.7** |
| **bvFTD** | **FTD-MND** | 0.9 | 6102.8 | 0.785 | -41898.8 | 49477.4 |
|  | **svPPA** | **-2.9** | **-30351.1** | **<.001** | **-41843.3** | **-18558.4** |
|  | **nfvPPA** | -0.5 | -11223.6 | 0.072 | -22863.8 | 1203.5 |
|  | **PPA-NOS** | **-2.7** | **-36336.6** | **<.001** | **-53296.3** | **-16613.6** |
| **FTD-MND** | **svPPA** | -3.8 | -36453.9 | 0.098 | -79816.9 | 13167.2 |
|  | **nfvPPA** | -1.4 | -17326.4 | 0.454 | -60481.7 | 31066.5 |
|  | **PPA-NOS** | -3.6 | -42439.4 | 0.050 | -85301.2 | 9657.2 |
| **svPPA** | **nfvPPA** | **2.3** | **19127.5** | **<.001** | **7118.2** | **30798.6** |
|  | **PPA-NOS** | 0.1 | -5985.5 | 0.488 | -22586.4 | 11652.5 |
| **nfvPPA** | **PPA-NOS** | **-2.2** | **-25113.0** | **0.008** | **-42964.3** | **-7544.3** |
| **Genetic diagnosis** | | | | | | |
| **Control** | ***C9orf72*** | **7.4** | **79618.3** | **<.001** | **56806.5** | **104109.6** |
|  | ***MAPT*** | **4.9** | **68201.5** | **<.001** | **52368.3** | **87151.7** |
|  | ***GRN*** | **9.5** | **104056.1** | **<.001** | **74820.3** | **142185.3** |
| ***C9orf72*** | ***MAPT*** | -2.7 | -11416.9 | 0.427 | -39589.9 | 17416.8 |
|  | ***GRN*** | 2.2 | 24437.8 | 0.226 | -14716.3 | 69778.8 |
| ***MAPT*** | ***GRN*** | 4.8 | 35854.6 | 0.056 | 2407.2 | 75425.9 |
| **Pathological diagnosis** | | | | | | |
| **Control** | **FUS** | **8.6** | **101651.7** | **0.001** | **65961.6** | **137989.7** |
|  | **TDP-43** | **5.4** | **57473.8** | **<.001** | **39691.2** | **78077.2** |
|  | **TAU** | **6.5** | **74975.8** | **<.001** | **61662.3** | **89306.6** |
| **FUS** | **TDP-43** | **-3.5** | **-44178.0** | **0.012** | **-81966.9** | **-5162.4** |
|  | **TAU** | -2.3 | -26676.0 | 0.099 | -61800.1 | 7441.5 |
| **TDP-43** | **TAU** | 1.2 | 17502.0 | 0.080 | -2526.1 | 35472.1 |

**Supplementary Table 5: Volumetric comparisons for the left and right hypothalamus between the clinical, genetic and pathological groups of patients and controls.** Volumes are expressed as percentage of TIV. Bold represents a significant difference between each FTD group and controls (reported p-values are adjusted for Bonferroni correction). SD denotes standard deviation. The % difference represents the volumetric difference between each FTD group and controls. Effect size values are partial eta squared.

|  |  | **Left hypothalamus** | | **Right hypothalamus** | |
| --- | --- | --- | --- | --- | --- |
| **Controls** | **Mean - SD** | 0.02948 | 0.00243 | 0.02817 | 0.00272 |
| **Clinical diagnosis** | | | | | |
| **bvFTD** | **Mean - SD** | 0.02482 | 0.00389 | 0.02358 | 0.00405 |
| **FTD-MND** | **Mean - SD** | 0.02348 | 0.00259 | 0.02260 | 0.00262 |
| **svPPA** | **Mean - SD** | 0.02435 | 0.00306 | 0.02470 | 0.00344 |
| **nfvPPA** | **Mean - SD** | 0.02692 | 0.00328 | 0.02604 | 0.00310 |
| **PPA-NOS** | **Mean - SD** | 0.02563 | 0.00369 | 0.02600 | 0.00299 |
| **% difference** | **bvFTD** | 16 | | 16 | |
|  | **FTD-MND** | 20 | | 20 | |
|  | **svPPA** | 17 | | 12 | |
|  | **nfvPPA** | 9 | | 8 | |
|  | **PPA-NOS** | 13 | | 8 | |
| **p-value** | **bvFTD** | **0.001** | | **0.001** | |
|  | **FTD-MND** | **0.001** | | **0.001** | |
|  | **svPPA** | **0.001** | | **0.001** | |
|  | **nfvPPA** | **0.001** | | **0.001** | |
|  | **PPA-NOS** | **0.001** | | **0.003** | |
| **effect size** | **bvFTD** | 0.217 | | 0.200 | |
|  | **FTD-MND** | 0.042 | | 0.032 | |
|  | **svPPA** | 0.055 | | 0.032 | |
|  | **nfvPPA** | 0.190 | | 0.080 | |
|  | **PPA-NOS** | 0.042 | | 0.010 | |
| **Genetic groups** | | | | | |
| ***C9orf72*** | **Mean - SD** | 0.02641 | 0.00399 | 0.02468 | 0.00457 |
| ***MAPT*** | **Mean - SD** | 0.02199 | 0.00395 | 0.02121 | 0.00422 |
| ***GRN*** | **Mean - SD** | 0.02580 | 0.00327 | 0.02508 | 0.00367 |
| **% difference** | ***C9orf72*** | 10 | | 12 | |
|  | ***MAPT*** | 25 | | 25 | |
|  | ***GRN*** | 12 | | 11 | |
| **p-value** | ***C9orf72*** | **0.001** | | **0.001** | |
|  | ***MAPT*** | **0.001** | | **0.001** | |
|  | ***GRN*** | **0.001** | | **0.001** | |
| **effect size** | ***C9orf72*** | 0.175 | | 0.170 | |
|  | ***MAPT*** | 0.520 | | 0.411 | |
|  | ***GRN*** | 0.151 | | 0.082 | |
| **Pathology groups** | | | | | |
| **FUS** | **Mean - SD** | 0.02019 | 0.00453 | 0.01864 | 0.00138 |
| **TDP-43** | **Mean - SD** | 0.02478 | 0.00310 | 0.02421 | 0.00316 |
| **Tau** | **Mean - SD** | 0.02277 | 0.00382 | 0.02249 | 0.00387 |
| **% difference** | **FUS** | 32 | | 34 | |
|  | **TDP-43** | 16 | | 14 | |
|  | **Tau** | 23 | | 20 | |
| **p-value** | **FUS** | **0.002** | | **0.001** | |
|  | **TDP-43** | **0.001** | | **0.001** | |
|  | **Tau** | **0.001** | | **0.001** | |
| **effect size** | **FUS** | 0.127 | | 0.130 | |
|  | **TDP-43** | 0.210 | | 0.136 | |
|  | **Tau** | 0.454 | | 0.339 | |
| **Pathology subgroups** | | | | | |
| **TDP-43 type A** | **Mean - SD** | 0.02614 | 0.00205 | 0.02541 | 0.00188 |
| **TDP-43 type B** | **Mean - SD** | 0.02393 | 0.00700 | 0.02255 | 0.00618 |
| **TDP-43 type C** | **Mean - SD** | 0.02386 | 0.00286 | 0.02353 | 0.00331 |
| **FTDP-17** | **Mean - SD** | 0.02199 | 0.00395 | 0.02121 | 0.00422 |
| **Tau Picks** | **Mean - SD** | 0.02301 | 0.00346 | 0.02296 | 0.00286 |
| **Tau CBD** | **Mean - SD** | 0.02547 | 0.00317 | 0.02575 | 0.00341 |
| **Tau PSP** | **Mean - SD** | 0.02350 | 0.00282 | 0.02332 | 0.00221 |
| **% difference** | **TDP-43 type A** | 11 | | 10 | |
|  | **TDP-43 type B** | 19 | | 20 | |
|  | **TDP-43 type C** | 19 | | 16 | |
|  | **FTDP17** | 25 | | 25 | |
|  | **Tau Pick’s** | 22 | | 19 | |
|  | **Tau CBD** | 14 | | 9 | |
|  | **Tau PSP** | 20 | | 17 | |
| **p-value** | **TDP-43 type A** | **0.001** | | **0.003** | |
|  | **TDP-43 type B** | **0.018** | | **0.029** | |
|  | **TDP-43 type C** | **0.001** | | **0.001** | |
|  | **FTDP17** | **0.001** | | **0.001** | |
|  | **Tau Pick’s** | **0.001** | | **0.001** | |
|  | **Tau CBD** | **0.049** | | 0.688 | |
|  | **Tau PSP** | **0.023** | | 0.077 | |
| **effect size** | **TDP-43 type A** | 0.073 | | 0.041 | |
|  | **TDP-43 type B** | 0.061 | | 0.056 | |
|  | **TDP-43 type C** | 0.210 | | 0.133 | |
|  | **FTDP17** | 0.502 | | 0.457 | |
|  | **Tau Pick’s** | 0.235 | | 0.142 | |
|  | **Tau CBD** | 0.028 | | 0.001 | |
|  | **Tau PSP** | 0.034 | | 0.013 | |

**Supplementary Table 6. Volumetric comparisons of the hypothalamic regions between the clinical groups and controls.** P-values are adjusted for Bonferroni correction. The mean difference represents the volumetric difference between each FTD group and the reference groups in mm^3^.

| **Clinical diagnosis** | | **a-iHyp** | | | | **a-sHyp** | | | | **infTub** | | | | **supTub** | | | | **posHyp** | | | | **Whole** | | | |
| --- | --- | --- | --- | --- | --- | --- | --- | --- | --- | --- | --- | --- | --- | --- | --- | --- | --- | --- | --- | --- | --- | --- | --- | --- | --- |
|  |  | **Mean difference** | **p-value** | **95% Confidence Interval** | | **Mean difference** | **p-value** | **95% Confidence Interval** | | **Mean difference** | **p-value** | **95% Confidence Interval** | | **Mean difference** | **p-value** | **95% Confidence Interval** | | **Mean difference** | **p-value** | **95% Confidence Interval** | | **Mean difference** | **p-value** | **95% Confidence Interval** | |
|  |  |  |  | **Lower** | **Upper** |  |  | **Lower** | **Upper** |  |  | **Lower** | **Upper** |  |  | **Lower** | **Upper** |  |  | **Lower** | **Upper** |  |  | **Lower** | **Upper** |
| **Control** | **bvFTD** | 10.476 | 0.001 | 8.553 | 12.360 | 14.088 | 0.001 | 12.605 | 15.559 | 13.849 | 0.001 | 7.618 | 19.739 | 36.469 | 0.001 | 30.881 | 42.061 | 45.896 | 0.001 | 38.508 | 53.543 | 120.779 | 0.001 | 103.087 | 137.437 |
|  | **FTD-MND** | 15.572 | 0.001 | 9.392 | 24.349 | 20.831 | 0.001 | 12.138 | 29.937 | 6.836 | 0.553 | -16.393 | 31.681 | 36.729 | 0.001 | 24.213 | 52.938 | 70.746 | 0.001 | 57.837 | 84.220 | 150.714 | 0.001 | 112.041 | 196.804 |
|  | **svPPA** | 9.853 | 0.001 | 7.754 | 11.909 | 12.394 | 0.001 | 10.609 | 14.272 | 18.519 | 0.001 | 11.851 | 25.275 | 34.475 | 0.001 | 27.676 | 41.024 | 30.204 | 0.001 | 22.398 | 38.385 | 105.445 | 0.001 | 87.274 | 123.972 |
|  | **nfvPPA** | 7.200 | 0.001 | 5.272 | 9.049 | 8.901 | 0.001 | 7.413 | 10.407 | 2.622 | 0.408 | -3.234 | 8.803 | 19.030 | 0.001 | 13.290 | 24.286 | 17.747 | 0.001 | 10.586 | 25.769 | 55.499 | 0.001 | 40.307 | 72.479 |
|  | **PPA-NOS** | 7.031 | 0.002 | 2.928 | 10.884 | 8.111 | 0.001 | 4.667 | 11.924 | 11.077 | 0.038 | 0.928 | 23.989 | 19.034 | 0.001 | 8.564 | 29.894 | 30.153 | 0.001 | 15.303 | 45.110 | 75.406 | 0.001 | 47.457 | 111.653 |
| **bvFTD** | **FTD-MND** | 5.096 | 0.174 | -1.081 | 14.285 | 6.743 | 0.101 | -1.876 | 15.672 | -7.013 | 0.554 | -30.825 | 17.643 | 0.259 | 0.965 | -12.261 | 17.116 | 24.850 | 0.001 | 11.580 | 38.407 | 29.935 | 0.142 | -11.335 | 78.349 |
|  | **svPPA** | -0.623 | 0.534 | -2.604 | 1.547 | -1.694 | 0.086 | -3.603 | 0.306 | 4.670 | 0.220 | -2.552 | 12.172 | -1.994 | 0.567 | -8.298 | 4.994 | -15.692 | 0.002 | -24.107 | -7.108 | -15.334 | 0.151 | -35.241 | 8.546 |
|  | **nfvPPA** | -3.276 | 0.001 | -5.173 | -1.392 | -5.188 | 0.001 | -6.767 | -3.561 | -11.227 | 0.004 | -18.221 | -3.408 | -17.440 | 0.001 | -22.985 | -11.923 | -28.150 | 0.001 | -36.232 | -19.957 | -65.280 | 0.001 | -84.303 | -46.637 |
|  | **PPA-NOS** | -3.445 | 0.092 | -7.682 | 0.572 | -5.978 | 0.001 | -9.748 | -2.053 | -2.772 | 0.649 | -13.149 | 10.954 | -17.435 | 0.002 | -27.817 | -6.328 | -15.744 | 0.041 | -31.310 | -0.628 | -45.374 | 0.011 | -75.820 | -8.532 |
| **FTD-MND** | **svPPA** | -5.719 | 0.132 | -15.129 | 0.542 | -8.437 | 0.040 | -17.384 | 0.235 | 11.683 | 0.340 | -13.500 | 34.869 | -2.253 | 0.767 | -18.909 | 10.720 | -40.542 | 0.001 | -55.388 | -26.123 | -45.269 | 0.022 | -91.307 | -5.097 |
|  | **nfvPPA** | -8.373 | 0.034 | -17.400 | -2.183 | -11.931 | 0.006 | -20.937 | -3.343 | -4.214 | 0.725 | -29.643 | 18.647 | -17.699 | 0.014 | -34.558 | -5.507 | -52.999 | 0.001 | -66.895 | -39.674 | -95.215 | 0.001 | -142.863 | -55.843 |
|  | **PPA-NOS** | -8.541 | 0.047 | -18.250 | -0.560 | -12.720 | 0.010 | -22.457 | -3.273 | 4.241 | 0.737 | -22.507 | 30.231 | -17.694 | 0.028 | -35.888 | -1.253 | -40.593 | 0.001 | -60.141 | -21.297 | -75.308 | 0.005 | -126.573 | -26.308 |
| **svPPA** | **nfvPPA** | -2.653 | 0.007 | -4.573 | -0.843 | -3.494 | 0.001 | -5.473 | -1.498 | -15.896 | 0.001 | -23.518 | -8.070 | -15.445 | 0.001 | -22.067 | -9.358 | -12.457 | 0.009 | -21.940 | -3.131 | -49.946 | 0.001 | -70.803 | -29.532 |
|  | **PPA-NOS** | -2.822 | 0.180 | -6.747 | 1.062 | -4.284 | 0.019 | -8.057 | -0.442 | -7.442 | 0.233 | -19.463 | 6.080 | -15.441 | 0.005 | -25.759 | -4.059 | -0.051 | 0.995 | -16.590 | 16.177 | -30.039 | 0.095 | -63.169 | 7.414 |
| **nfvPPA** | **PPA-NOS** | -0.169 | 0.928 | -4.501 | 3.631 | -0.790 | 0.672 | -4.458 | 3.222 | 8.455 | 0.189 | -2.843 | 22.467 | 0.004 | 1.000 | -10.198 | 11.052 | 12.406 | 0.097 | -2.964 | 27.258 | 19.907 | 0.231 | -10.978 | 57.163 |

**Supplementary Table 7. Volumetric comparisons of the hypothalamic regions between the genetic groups and controls.** P-values are adjusted for Bonferroni correction. The mean difference represents the volumetric difference between each FTD group and the reference groups in mm^3^.

| **Genetic groups** | | **a-iHyp** | | | | **a-sHyp** | | | | **infTub** | | | | **supTub** | | | | **posHyp** | | | | **Whole** | | | |
| --- | --- | --- | --- | --- | --- | --- | --- | --- | --- | --- | --- | --- | --- | --- | --- | --- | --- | --- | --- | --- | --- | --- | --- | --- | --- |
|  |  | **Mean difference** | **p-value** | **95% Confidence Interval** | | **Mean difference** | **p-value** | **95% Confidence Interval** | | **Mean difference** | **p-value** | **95% Confidence Interval** | | **Mean difference** | **p-value** | **95% Confidence Interval** | | **Mean difference** | **p-value** | **95% Confidence Interval** | | **Mean difference** | **p-value** | **95% Confidence Interval** | |
|  |  |  |  | **Lower** | **Upper** |  |  | **Lower** | **Upper** |  |  | **Lower** | **Upper** |  |  | **Lower** | **Upper** |  |  | **Lower** | **Upper** |  |  | **Lower** | **Upper** |
| **Control** | ***C9orf72*** | 10.699 | 0.001 | 6.946 | 14.617 | 13.917 | 0.001 | 10.155 | 17.134 | 0.148 | 0.979 | -12.431 | 12.998 | 27.799 | 0.001 | 16.182 | 38.704 | 40.200 | 0.001 | 24.293 | 53.979 | 92.764 | 0.001 | 59.146 | 129.052 |
|  | ***MAPT*** | 15.095 | 0.001 | 11.723 | 18.432 | 20.633 | 0.001 | 18.141 | 23.461 | 30.268 | 0.001 | 16.446 | 44.304 | 61.117 | 0.001 | 49.190 | 73.078 | 82.917 | 0.001 | 70.737 | 94.831 | 210.030 | 0.001 | 175.113 | 243.812 |
|  | ***GRN*** | 12.328 | 0.001 | 9.383 | 15.620 | 12.828 | 0.001 | 8.660 | 16.581 | -7.236 | 0.315 | -20.920 | 7.443 | 18.073 | 0.003 | 7.980 | 29.387 | 48.657 | 0.001 | 31.180 | 68.004 | 84.650 | 0.001 | 50.304 | 124.590 |
| ***C9orf72*** | ***MAPT*** | 4.395 | 0.062 | -0.612 | 8.649 | 6.716 | 0.005 | 2.405 | 11.604 | 30.120 | 0.001 | 12.750 | 48.448 | 33.318 | 0.001 | 18.870 | 48.315 | 42.717 | 0.001 | 24.274 | 60.287 | 117.266 | 0.001 | 73.932 | 166.560 |
|  | ***GRN*** | 1.629 | 0.505 | -2.952 | 6.574 | -1.089 | 0.661 | -6.623 | 3.921 | -7.384 | 0.410 | -25.579 | 10.957 | -9.726 | 0.184 | -22.978 | 4.758 | 8.457 | 0.486 | -12.090 | 32.720 | -8.114 | 0.737 | -55.119 | 45.029 |
| ***MAPT*** | ***GRN*** | -2.767 | 0.236 | -6.775 | 1.848 | -7.805 | 0.003 | -12.599 | -3.113 | -37.504 | 0.001 | -55.875 | -19.500 | -43.044 | 0.001 | -56.745 | -27.213 | -34.260 | 0.003 | -53.946 | -13.464 | -125.379 | 0.001 | -168.738 | -73.472 |

**Supplementary Table 8. Volumetric comparisons of the hypothalamic regions between the primary pathology groups and controls.** P-values are adjusted for Bonferroni correction. The mean difference represents the volumetric difference between each FTD group and the reference groups in mm^3^.

| **Pathology groups** | | **a-iHyp** | | | | **a-sHyp** | | | | **infTub** | | | | **supTub** | | | | **posHyp** | | | | **Whole** | | | |
| --- | --- | --- | --- | --- | --- | --- | --- | --- | --- | --- | --- | --- | --- | --- | --- | --- | --- | --- | --- | --- | --- | --- | --- | --- | --- |
|  |  | **Mean difference** | **p-value** | **95% Confidence Interval** | | **Mean difference** | **p-value** | **95% Confidence Interval** | | **Mean difference** | **p-value** | **95% Confidence Interval** | | **Mean difference** | **p-value** | **95% Confidence Interval** | | **Mean difference** | **p-value** | **95% Confidence Interval** | | **Mean difference** | **p-value** | **95% Confidence Interval** | |
|  |  |  |  | **Lower** | **Upper** |  |  | **Lower** | **Upper** |  |  | **Lower** | **Upper** |  |  | **Lower** | **Upper** |  |  | **Lower** | **Upper** |  |  | **Lower** | **Upper** |
| **Control** | **FUS** | 21.150 | 0.001 | 10.051 | 30.925 | 22.634 | 0.001 | 10.373 | 32.768 | 24.070 | 0.278 | -36.889 | 59.985 | 64.061 | 0.001 | 31.738 | 102.167 | 94.608 | 0.001 | 80.944 | 109.569 | 226.524 | 0.001 | 110.036 | 323.213 |
|  | **TDP-43** | 10.415 | 0.001 | 8.015 | 12.956 | 12.415 | 0.001 | 9.625 | 15.295 | 6.957 | 0.217 | -4.657 | 18.691 | 29.720 | 0.001 | 21.081 | 38.350 | 38.214 | 0.001 | 27.901 | 49.041 | 97.722 | 0.001 | 74.153 | 124.250 |
|  | **TAU** | 13.055 | 0.001 | 10.232 | 15.569 | 17.356 | 0.001 | 14.777 | 19.913 | 17.911 | 0.002 | 7.512 | 29.465 | 45.476 | 0.001 | 36.096 | 54.829 | 59.964 | 0.001 | 49.452 | 71.474 | 153.762 | 0.001 | 125.048 | 184.869 |
| **FUS** | **TDP-43** | -10.735 | 0.012 | -20.773 | 0.247 | -10.219 | 0.028 | -21.457 | 1.760 | -17.113 | 0.462 | -52.501 | 42.937 | -34.341 | 0.022 | -72.112 | -1.762 | -56.394 | 0.001 | -73.244 | -39.360 | -128.803 | 0.008 | -226.043 | -11.294 |
|  | **TAU** | -8.095 | 0.068 | -17.951 | 3.137 | -5.279 | 0.270 | -16.289 | 6.841 | -6.159 | 0.813 | -42.420 | 57.587 | -18.585 | 0.251 | -57.599 | 13.045 | -34.644 | 0.001 | -50.654 | -18.996 | -72.762 | 0.121 | -169.015 | 47.202 |
| **TDP-43** | **TAU** | 2.640 | 0.089 | -0.549 | 5.629 | 4.941 | 0.008 | 1.322 | 8.524 | 10.954 | 0.123 | -2.847 | 25.006 | 15.756 | 0.003 | 5.470 | 26.790 | 21.750 | 0.002 | 8.102 | 35.978 | 56.041 | 0.001 | 21.887 | 88.156 |

**Supplementary Table 9. Volumetric comparisons for the hypothalamic nuclei between the specific pathology subgroups and controls.** P-values are adjusted for Bonferroni correction. The mean difference represents the volumetric difference between each FTD group and the reference groups in mm^3^.

| **Subpathology groups** | | **a-iHyp** | | | | **a-sHyp** | | | | **infTub** | | | | **supTub** | | | | **posHyp** | | | | **Whole** | | | |
| --- | --- | --- | --- | --- | --- | --- | --- | --- | --- | --- | --- | --- | --- | --- | --- | --- | --- | --- | --- | --- | --- | --- | --- | --- | --- |
|  |  | **Mean Difference** | **p-value** | **95% Confidence Interval** | | **Mean Difference** | **p-value** | **95% Confidence Interval** | | **Mean Difference** | **p-value** | **95% Confidence Interval** | | **Mean Difference** | **p-value** | **95% Confidence Interval** | | **Mean Difference** | **p-value** | **95% Confidence Interval** | | **Mean Difference** | **p-value** | **95% Confidence Interval** | |
|  |  |  |  | **Lower** | **Upper** |  |  | **Lower** | **Upper** |  |  | **Lower** | **Upper** |  |  | **Lower** | **Upper** |  |  | **Lower** | **Upper** |  |  | **Lower** | **Upper** |
| **Control** | **TDP-43 type A** | 10.212 | 0.001 | 6.173 | 13.932 | 12.268 | 0.001 | 7.084 | 16.831 | -10.118 | 0.149 | -24.906 | 3.027 | 14.640 | 0.002 | 6.396 | 24.197 | 37.844 | 0.001 | 21.429 | 54.299 | 64.846 | 0.001 | 37.892 | 89.818 |
|  | **TDP-43 type B** | 10.092 | 0.001 | 3.464 | 20.104 | 16.293 | 0.001 | 8.552 | 26.756 | 27.378 | 0.190 | -8.19 | 76.323 | 40.554 | 0.005 | 16.204 | 76.482 | 51.877 | 0.030 | 14.571 | 110.597 | 146.194 | 0.027 | 43.498 | 307.276 |
|  | **TDP-43 type C** | 10.323 | 0.001 | 7.695 | 13.213 | 11.667 | 0.001 | 8.192 | 15.176 | 15.924 | 0.038 | 0.508 | 31.254 | 38.462 | 0.001 | 27.347 | 49.334 | 33.264 | 0.001 | 19.250 | 47.180 | 109.640 | 0.001 | 79.578 | 144.319 |
|  | **FTDP17** | 14.450 | 0.001 | 11.157 | 17.856 | 19.867 | 0.001 | 17.035 | 22.644 | 32.083 | 0.001 | 17.260 | 45.177 | 59.906 | 0.001 | 47.875 | 72.071 | 80.898 | 0.001 | 67.910 | 92.181 | 207.204 | 0.001 | 172.569 | 239.841 |
|  | **Tau Pick’s** | 12.190 | 0.001 | 8.967 | 15.514 | 17.947 | 0.001 | 13.116 | 23.328 | 16.695 | 0.006 | 5.104 | 28.686 | 40.585 | 0.001 | 29.475 | 52.405 | 42.806 | 0.001 | 29.614 | 57.048 | 130.224 | 0.001 | 96.246 | 162.309 |
|  | **Tau CBD** | 9.501 | 0.006 | 3.389 | 15.446 | 7.039 | 0.004 | 2.661 | 12.575 | -16.250 | 0.139 | -36.213 | 7.650 | 15.189 | 0.060 | 0.664 | 30.187 | 19.131 | 0.079 | -0.722 | 41.810 | 34.609 | 0.201 | -24.239 | 95.609 |
|  | **Tau PSP** | 7.049 | 0.353 | -9.297 | 20.502 | 13.623 | 0.001 | 8.971 | 18.109 | -5.846 | 0.459 | -23.291 | 10.86 | 19.566 | 0.034 | -3.107 | 39.972 | 43.876 | 0.036 | -8.664 | 79.552 | 78.267 | 0.042 | -27.587 | 153.064 |
| **TDP-43 type A** | **TDP-43 type B** | -0.119 | 0.977 | -8.26 | 10.318 | 4.025 | 0.486 | -5.577 | 16.112 | 37.496 | 0.067 | 0.128 | 88.077 | 25.914 | 0.086 | 0.454 | 62.409 | 14.033 | 0.618 | -27.704 | 74.995 | 81.348 | 0.264 | -21.026 | 246.808 |
|  | **TDP-43 type C** | 0.112 | 0.958 | -3.847 | 4.539 | -0.601 | 0.851 | -6.135 | 5.493 | 26.042 | 0.013 | 5.892 | 45.750 | 23.822 | 0.001 | 10.698 | 35.778 | -4.580 | 0.645 | -23.434 | 16.063 | 44.794 | 0.025 | 7.843 | 84.390 |
|  | **FTDP17** | 4.239 | 0.080 | -0.236 | 9.231 | 7.599 | 0.007 | 2.432 | 13.464 | 42.201 | 0.001 | 22.974 | 59.598 | 45.266 | 0.001 | 30.883 | 59.975 | 43.054 | 0.001 | 25.140 | 60.856 | 142.359 | 0.001 | 103.120 | 183.845 |
|  | **Tau Pick’s** | 1.978 | 0.390 | -2.620 | 6.886 | 5.679 | 0.112 | -0.786 | 13.336 | 26.813 | 0.005 | 10.444 | 43.253 | 25.945 | 0.001 | 13.370 | 37.603 | 4.962 | 0.601 | -13.029 | 24.744 | 65.378 | 0.004 | 25.181 | 104.633 |
|  | **Tau CBD** | -0.711 | 0.848 | -7.383 | 6.152 | -5.229 | 0.122 | -11.784 | 1.544 | -6.132 | 0.610 | -31.512 | 20.256 | 0.549 | 0.937 | -15.029 | 15.706 | -18.713 | 0.127 | -42.602 | 6.428 | -30.236 | 0.305 | -88.430 | 33.124 |
|  | **Tau PSP** | -3.163 | 0.661 | -19.163 | 10.981 | 1.355 | 0.675 | -4.573 | 7.68 | 4.272 | 0.705 | -16.935 | 26.476 | 4.926 | 0.656 | -17.253 | 25.301 | 6.032 | 0.822 | -46.835 | 45.609 | 13.422 | 0.765 | -89.279 | 91.331 |
| **TDP-43 type B** | **TDP-43 type C** | 0.231 | 0.966 | -9.663 | 7.245 | -4.626 | 0.376 | -15.650 | 3.778 | -11.454 | 0.665 | -63.225 | 26.945 | -2.092 | 0.921 | -40.177 | 24.035 | -18.613 | 0.503 | -78.612 | 22.430 | -36.554 | 0.683 | -202.482 | 75.347 |
|  | **FTDP17** | 4.358 | 0.327 | -5.668 | 11.374 | 3.574 | 0.503 | -7.202 | 11.808 | 4.705 | 0.871 | -48.668 | 42.727 | 19.352 | 0.262 | -17.732 | 47.829 | 29.021 | 0.264 | -31.013 | 68.451 | 61.011 | 0.422 | -110.320 | 169.462 |
|  | **Tau Pick’s** | 2.098 | 0.688 | -7.933 | 9.285 | 1.654 | 0.783 | -9.512 | 11.538 | -10.682 | 0.687 | -59.842 | 26.015 | 0.032 | 0.999 | -36.662 | 26.864 | -9.071 | 0.776 | -68.381 | 29.862 | -15.970 | 0.865 | -183.493 | 90.309 |
|  | **Tau CBD** | -0.591 | 0.922 | -11.822 | 8.721 | -9.254 | 0.061 | -20.477 | -0.164 | -43.628 | 0.057 | -98.188 | -0.538 | -25.365 | 0.118 | -63.686 | 4.120 | -32.746 | 0.220 | -92.574 | 11.518 | -111.585 | 0.122 | -279.283 | 12.055 |
|  | **Tau PSP** | -3.043 | 0.711 | -18.970 | 14.522 | -2.670 | 0.604 | -13.883 | 6.777 | -33.224 | 0.144 | -82.874 | 9.054 | -20.988 | 0.260 | -62.787 | 14.139 | -8.001 | 0.834 | -77.526 | 53.570 | -67.926 | 0.426 | -243.845 | 72.273 |
| **TDP-43 type C** | **FTDP17** | 4.127 | 0.025 | 0.308 | 7.678 | 8.200 | 0.001 | 3.821 | 12.028 | 16.159 | 0.129 | -4.989 | 35.162 | 21.444 | 0.010 | 6.215 | 37.388 | 47.634 | 0.001 | 30.043 | 63.236 | 97.564 | 0.001 | 52.073 | 142.201 |
|  | **Tau Pick’s** | 1.866 | 0.308 | -1.785 | 5.358 | 6.280 | 0.036 | 0.595 | 12.299 | 0.772 | 0.929 | -15.990 | 18.190 | 2.124 | 0.773 | -12.018 | 16.790 | 9.542 | 0.281 | -7.729 | 27.390 | 20.583 | 0.337 | -23.047 | 62.595 |
|  | **Tau CBD** | -0.822 | 0.791 | -6.836 | 5.729 | -4.629 | 0.112 | -10.252 | 1.247 | -32.174 | 0.013 | -55.892 | -5.478 | -23.273 | 0.013 | -39.715 | -5.973 | -14.133 | 0.209 | -36.721 | 9.700 | -75.031 | 0.018 | -139.423 | -11.493 |
|  | **Tau PSP** | -3.275 | 0.621 | -20.007 | 10.279 | 1.955 | 0.471 | -3.197 | 7.363 | -21.770 | 0.041 | -42.816 | -1.504 | -18.896 | 0.079 | -43.343 | 3.232 | 10.612 | 0.683 | -39.553 | 48.578 | -31.373 | 0.475 | -134.183 | 48.999 |
| **FTDP-17** | **Tau Pick’s** | -2.261 | 0.235 | -6.032 | 1.451 | -1.920 | 0.475 | -6.928 | 3.783 | -15.388 | 0.063 | -30.469 | 0.942 | -19.321 | 0.011 | -34.102 | -3.376 | -38.092 | 0.001 | -53.435 | -21.390 | -76.981 | 0.003 | -121.646 | -31.970 |
|  | **Tau CBD** | -4.949 | 0.135 | -11.378 | 1.901 | -12.828 | 0.001 | -17.556 | -7.258 | -48.333 | 0.001 | -69.546 | -22.321 | -44.717 | 0.001 | -62.316 | -26.163 | -61.767 | 0.001 | -83.765 | -37.827 | -172.595 | 0.001 | -237.315 | -104.824 |
|  | **Tau PSP** | -7.402 | 0.303 | -24.266 | 6.930 | -6.244 | 0.011 | -11.545 | -1.244 | -37.929 | 0.001 | -59.286 | -16.387 | -40.340 | 0.001 | -63.198 | -18.658 | -37.022 | 0.087 | -90.717 | 1.491 | -128.937 | 0.003 | -235.430 | -42.555 |
| **Tau Pick's** | **Tau CBD** | -2.689 | 0.411 | -9.164 | 3.595 | -10.908 | 0.002 | -17.453 | -4.771 | -32.946 | 0.009 | -56.501 | -8.681 | -25.396 | 0.005 | -42.396 | -8.161 | -23.675 | 0.034 | -46.525 | -2.616 | -95.614 | 0.001 | -159.381 | -32.280 |
|  | **Tau PSP** | -5.141 | 0.445 | -21.050 | 8.972 | -4.324 | 0.174 | -11.080 | 2.323 | -22.542 | 0.022 | -43.429 | -2.405 | -21.019 | 0.049 | -45.623 | 2.399 | 1.071 | 0.972 | -49.692 | 39.193 | -51.956 | 0.221 | -154.482 | 31.170 |
| **Tau CBD** | **Tau PSP** | -2.452 | 0.738 | -19.007 | 12.929 | 6.584 | 0.026 | 0.079 | 12.770 | 10.404 | 0.382 | -19.533 | 36.465 | 4.377 | 0.694 | -19.742 | 26.633 | 24.745 | 0.282 | -29.786 | 69.195 | 43.658 | 0.385 | -61.542 | 139.382 |

**Supplementary Figure 1: Segmentation of the hypothalamic subunits mapped on a 3T T1-weighted MR image of a control subject.** Abbreviations: anterior inferior hypothalamus (a-iHyp), anterior superior hypothalamus (a-sHyp), tubular inferior hypothalamus (infTub), tubular superior hypothalamus (supTub), posterior hypothalamus (posHyp).

**
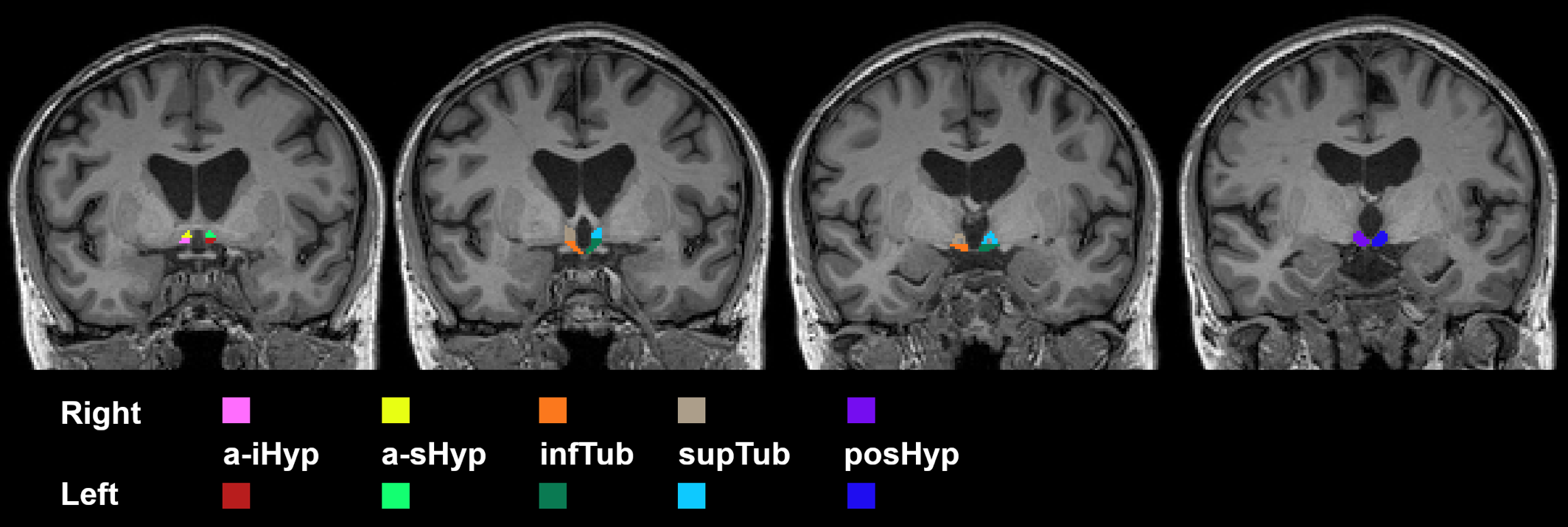
**

**Supplementary Figure 2: Scatterplots for the clinical (A), genetic (B) and pathology (C) groups for the CBI-R eating behavioural scores.**


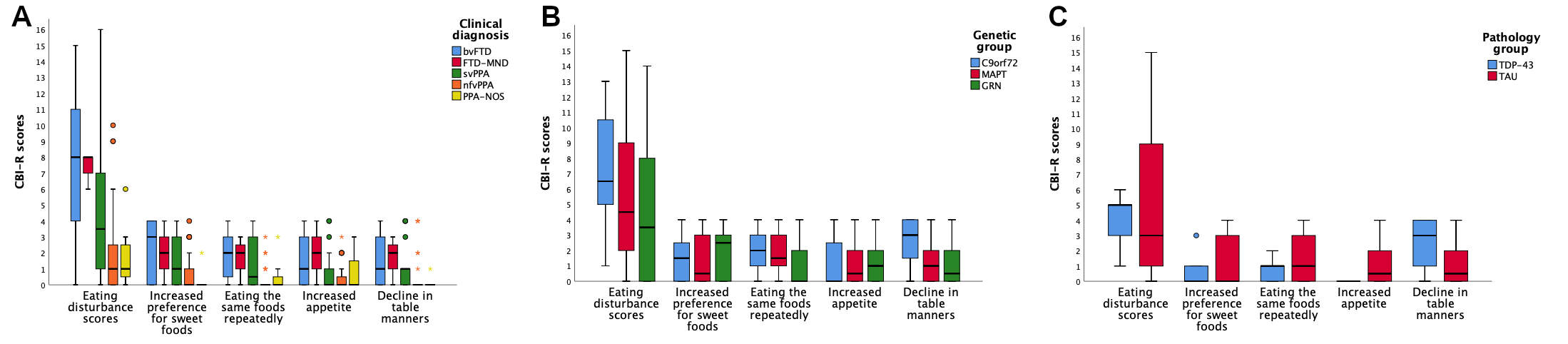


**Supplementary Figure 3: Scatterplots for the clinical (A), genetic (B) and pathology (C) groups for the hypothalamic regions expressed as percentage of total intracranial volume.** Abbreviations: anterior inferior hypothalamus (a-iHyp), anterior superior hypothalamus (a-sHyp), tubular inferior hypothalamus (infTub), tubular superior hypothalamus (supTub), posterior hypothalamus (posHyp).

**
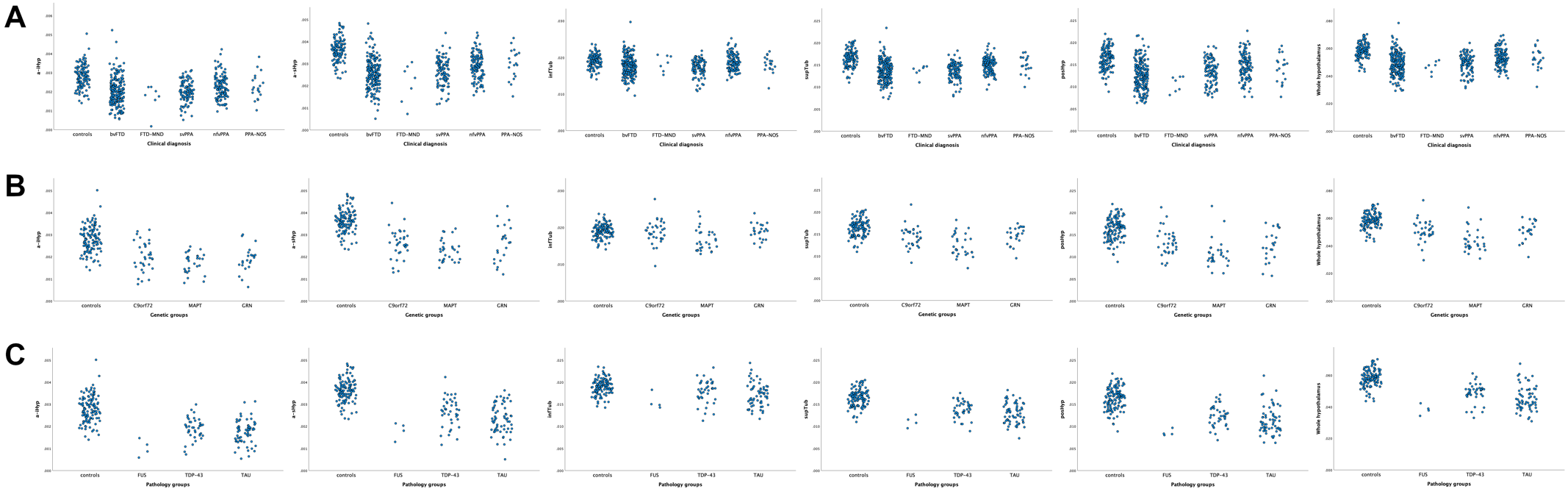
**
